# Siloxane Decorated Water‐Obstructing Guest for Efficient Air‐Processed OSCs

**DOI:** 10.1002/advs.202412190

**Published:** 2025-02-20

**Authors:** Yurong He, Wentao Miao, Tianyu Hu, Junchi Su, Aziz Saparbaev, Ming Wan, Jingnan Wu, Yuda Li, Huimin Xiang, Ergang Wang, Xunchang Wang, Renqiang Yang

**Affiliations:** ^1^ Key Laboratory of Optoelectronic Chemical Materials and Devices (Ministry of Education), School of Optoelectronic Materials & Technology Jianghan University Wuhan 430056 China; ^2^ Institute of Ion‐plasma and Laser Technologies National University of Uzbekistan Tashkent 100174 Uzbekistan; ^3^ Department of Chemistry and Chemical Engineering Chalmers University of Technology Göteborg 41296 Sweden; ^4^ Key Laboratory of Novel Biomass‐based Environmental and Energy Materials in Petroleum and Chemical Industry School of Chemical Engineering and Pharmacy Wuhan Institute of Technology Wuhan 430205 China

**Keywords:** air‐processed organic solar cell, high humidity, siloxane, water‐obstructing guest

## Abstract

The future applications of organic solar cells (OSCs) necessitate a thorough consideration of their ambient stability and processability, particularly for large area air‐processed engineering, but water‐induced degradation of active layer critically restricts its development. To surmount this hurdle, a water‐obstructing guest (WOG) strategy is proposed to attenuate the interaction of the active layer with water molecules, reduce defects in blend films, and enhance the devices stability under high relative humidity (RH) conditions by introducing a siloxane‐containing polymer D18‐SiO. In addition to suppressing trap density, the WOG with hydrophobic and low surface free energy characteristics, forms a capping layer that blocks moisture penetration while preserving ideal nano‐micromorphology with high crystallinity and tight packing properties. Power conversion efficiencies (PCE) of >19% is reported for spin coating OSCs fabricated across an RH range of 20 to 90%, and PCE of >17% blade coating OSCs at 90% RH. The D18‐SiO, serves as a protective barrier to enhance the device stability, and the corresponding unencapsulated OSCs retained 80.7% of its initial performance in air (≈ 40% RH) after 600‐h maximum power point tracking under continuous light illumination, showcasing great potential in designing WOG strategy for large‐scale production of air‐processed OSCs.

## Graphical abstract

The Water‐obstructing Guest (WOG) strategy, employing a siloxane decorated polymer D18‐SiO as a capping layer, is proposed to minimize moisture penetration while preserving original morphology and suppresing trap density in high humidity condition. WOG‐based Organic solar cells, fabricated at 90% relative humidity (RH), achieved a power conversion efficiency (PCE) of 19.1% with excellent stability.

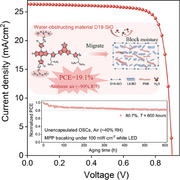

## 1 Introduction

Organic solar cell (OSC) has emerged as an environmentally friendly and sustainable energy convention technology with the power conversion efficiency (PCE) soaring from 11% to over 20% in recent 10 years.^[^ ^1^, ^2^, ^3^, ^4^, ^5^, ^6^, ^7^, ^8^, ^9^, ^10^ ^]^ To data, the majority of advancements in OSCs with high PCE have been made by spin coating in a protective nitrogen atmosphere, which is not scalable for the large‐scale manufacturing of solar panels. Although air‐processed OSCs are recognized essential for low‐cost commercial modulus, the fabricating high‐quality OSCs in ambient condition remains a significant challenge.^[^ ^11^, ^12^, ^13^, ^14^ ^]^ Previous work has reported that moisture in the air consistently leads to deteriorated active layers and further influences the crystallographic quality and defect density.^[^ ^15^, ^16^ ^]^ The mechanism was proposed that when the water infiltrate into the active layer, the water molecules or clusters have significantly lowered ionization energies and hence can form potential electron or hole traps, supported by recent finding from electron‐only and hole‐only diodes made of several organic photovoltaic materials.^[^ ^17^, ^18^ ^]^ The water‐induced traps can further lead to diminished crystallinity and an imbalance in carrier mobility, which are more likely to form intermolecular voids within the disordered conjugated materials microstructure, causing unavoidable non‐radiative recombination and dramatically reduced quantum efficiency.^[^ ^19^, ^20^ ^]^ Therefore, finding an effective strategy to avoid water molecules into the blend morphology for air‐processed OSCs as much as possible is necessary, which will maintain the excellent device performance and long‐term stability, and attract more attention from the scientific and industrial communities.

Several studies have been conducted to eliminate the damage of moisture to devices, aiming to improve the humidity tolerance for ambient processing of OSCs.^[^ ^21^, ^22^, ^23^, ^24^, ^25^, ^26^, ^27^ ^]^ For example, the perylene imide polymer with high planar backbone and high electron mobility, PDI‐V, were developed as acceptor to dramatically improve crystallization of active layers and strongly strength the humidity tolerance.^[^ ^28^ ^]^ The device processed in an air environment with 90% humidity can reach a high PCE of 7.49%, which is reduced by only 1% compared with the device prepared in glove boxes. Zhang et al. prepared OSCs by developing a series of benzo‐ [1,2b:4,5‐b 2] furan polymers at 70–80% humidity, and promising ambient stability even after ≈ 800–1000 h of air aging were achieved,^[^ ^29^ ^]^ with PCEs exhibiting only 0.3‐2% loss compared to those of the devices from inert condition. Despite the advancements made in the development of active layer materials for OSCs processed at high‐humidity environments, the device efficiency remains inferior to that of state‐of‐the‐art organic solar cells (OSCs) processed in N_2_ atmosphere.^[^ ^30^, ^31^ ^]^ Recently, Chen et al. demonstrated that the hydrophobic characteristics of siloxane terminated side chains of polymer PQSi705 can mitigate the moisture condensation on the blend film surface even under high humidity conditions and suppress the penetration of water droplets into the blend film,^[^ ^32^ ^]^ thereby restraining water induced morphology damage to the blend film. As a result, the PQSi705:m‐TEH active layers processed in the N_2_ glovebox and 90% RH air exhibited nearly identical PCEs of ≈ 18%. Unfortunately, although siloxane side chain induced high humidity tolerance for ambient processing of OSCs has promoted the rapid development of PCE,^[^ ^33^, ^34^, ^35^, ^36^ ^]^ the depth mechanism of resistance to humidity still needs more comprehensive analysis.^[^ ^37^, ^38^, ^39^, ^40^ ^]^ Furthermore, a fundamental understanding of specific features brought by the siloxane side chain modification in terms of the distribution of hydrophobic group, morphology evolution, trap density, carrier dynamics, and device stability is still lacking, which can be highly desirable to accelerate the development of highly efficient air‐processed OSCs with high humidity tolerance.

In this contribution, we designed and utilized a new siloxane terminated side chains‐based polymer D18‐SiO as the third component,^[^ ^41^ ^]^ and reveal how this siloxane decoration guest affects the processability, stability, and overall performance of the state‐of‐art OSCs in high‐humidity conditions. The siloxane‐based polymer with hydrophobic features tends to aggregate on the film surface during the solution processing in air, and spontaneously migrate to the solution‐air interface and self‐confine to a capping layer. The formed capping layer acts as a waterproof coating, which suppress the penetration of water droplets into the blend film under high humidity conditions, and thereby mitigating water‐induced morphological damage. In addition, the siloxane‐based polymer guest endowed blend film morphology with maintained excellent interpenetrating network micro‐nano structure, which could inhibit large‐scale molecular aggregation during long‐term placement in air and keep its tight molecular packing from being destroyed, thus minimize the generation of defect modes, and meanwhile sustaining the efficiency of charge transfer/separation. One the basis of these guest molecules, we propose a water‐obstructing guest (WOG) strategy, which markedly facilitate the self‐migration to the periphery through surface energy discrepancy of photovoltaic materials, effectively passivates the trap density by maintaining morphology, and functions as a protective barrier to shield the devices from external moisture intrusion. Eventually, the WOG strategy efficiency enables impressive efficiency >19% and 17% for spin coating and blade coating devices at 90% RH, which record one of the highest values under high‐humidity treatment. Remarkably, robust light stability is also obtained, in which the device with WOG strategy in open‐air (≈ 40% RH) maintains ≈ 80.7% of its initial PCE after 600 h of operation at the maximum power point, showcasing great potential in designing water‐obstructing polymer for the future application of air‐processed OSCs.

## 2 Results and Discussion

### 2.1 Material Characterization of Water‐Obstructing Guest and the Impact of WOG on the Device Performance

**Figure 1:**
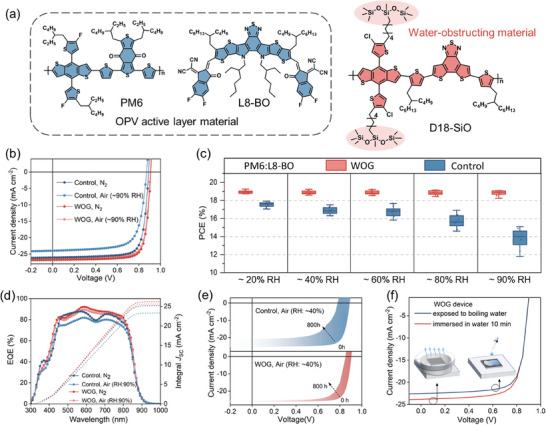
a) Chemical structures of PM6 and L8‐BO, and the water‐obstructing material D18‐SiO. b) *J–V* curves of PM6: L8‐BO and PM6: D18‐SiO: L8‐BO based devices. c) Device efficiency distribution at different humidity. d) The corresponding EQE curves. e) *J‐V* curves control and WOG devices after 800 h storage. f) *J‐V* curves of WOG device under different water conditions.

The chemical structures of the polymer donor PM6, the small molecular acceptor L8‐BO, and the new synthesized water‐obstructing polymer, namely, D18‐SiO which featured with siloxane terminated side chains were shown in **Figure** **1**a. The synthetic routes of the D18‐SiO, the corresponding nuclear magnetic resonance spectra, the number‐average molecular weight and polydispersity index were displayed in Supporting Information. D18‐SiO show good solubility in common solvents, such as chlorobenzene and chloroform. As shown in Figure S2 (Supporting Information), D18‐SiO exhibits absorption ranging from 450 ≈ 600 nm, which forms complementary absorption with PM6 and L8‐BO. Therefore, the ternary systems could cover a wide range of solar spectrum to harvest more photons. The frontier energy levels of these active materials were presented in Figure S3 (Supporting Information). D18‐SiO exhibits the highest occupied molecular orbital (HOMO) and the lowest unoccupied molecular orbital of −5.89 and −3.94 eV, lying between those energy levels of PM6 and L8‐BO. The cascade HOMO energy level alignment formed in such a ternary blend is conducive to producing high *V*_OC_ and facilitating charge transfer and extraction in the corresponding device.^[^ ^42^, ^43^, ^44^ ^]^

The devices processed in open air with RH of 90% and nitrogen atmosphere were fabricated to investigate the impact of WOG strategy on photovoltaic performance by adopting the conventional device of ITO/PEDOT: PSS/active layer/PDINN/Ag. The ternary blends with the best ratio for PM6: D18‐SiO: L8‐BO (WOG) is 1:0.03:1.2, and the corresponding photovoltaic parameters are shown in Figure 1b and **Table** **1**. The controlled OSCs processed under N_2_ atmosphere delivered a PCE of 18.3%, with an open‐circuit voltage (*V*_OC_) of 0.889 V, short‐circuit current density (*J*_SC_) of 26.0 mA cm^−2^, fill factor (FF) of 79.2%. However, this device suffered a dramatically decline in performance when processed in open‐air with ≈ 90%RH, with an inferior PCE of 15.1%. In contrast, the WOG OSCs prepared under two different conditions exhibit little difference in performance, with both higher PCEs of over 19%. Specifically, WOG endows the best PCE of 19.1% for OSCs processed in open‐air (RH:90%), entailing a *V*_OC_ of 0.904 V, a *J*_SC_ of 26.4 mA cm^−2^ and an FF of 80.2%, as shown in Table 1. The noteworthy aspect lies in the fact that this represents the pinnacle of PCE among photovoltaic cells processed at ambient atmosphere, thereby showcasing the inherent potential value of the proposed WOG strategy based on siloxane terminated side chains‐containing polymer guest within the field of OSCs. As a means of verifying *J*_SC_, Figure 1d exhibits the external quantum efficiency (EQE) curves of the control and WOG based OSCs. The integrated *J*_SC_ values extracted from EQE curves are consistent with that of *J‐V* results with acceptable nuance (<3%) (see Table 1). Meanwhile, we also prepared inverted devices with the structure of ITO/ZnO/active layer/MoO_3_/Ag. The inverse device has a similar change trend with the conventional one that higher PCE of 18.1% can be observed in WOG devices (Table S2, Supporting Information).

**Table 1:** Photovoltaic parameters of the conventional OSCs processed with and without WOG under the illumination of AM 1.5G (100 mW cm^−2^).

Active layer (PM6:L8‐BO)	Fabricating Condition	*V*_OC_[V]	FF[%]	*J*_SC_[mA/cm^2^]	*J*_cal_[mA/cm^2^] ^a)^	PCE[%] ^b)^
Control	N_2_	0.889	79.2	26.0	25.3	18.3 (17.9 ± 0.4)
Control	Air (RH:90%)	0.869	72.7	23.9	23.4	15.1 (13.1 ± 2.1)
WOG	N_2_	0.907	80.7	26.8	26.1	19.6 (19.2 ± 0.4)
WOG	Air (RH:90%)	0.904	80.2	26.4	25.7	19.1 (18.5 ± 0.6)

^a)^ The *J*_cal_ represents the integrated current density obtained from EQE spectra;

^b)^ The average values and standard deviations of 15 devices are shown in parentheses.

Reproducible OSCs under varying high‐humidity ambient conditions (20 ≈ 90% RH) are a prerequisite for their commercialization. We observed that the control device is highly sensitive to humidity levels: The average PCE decreased from 17.4% to 15.2% as the RH increased from 20% to 80%, accompanied by broader PCE distributions (Figure 1c). Dramatically reduced PCE (lower than 3%) was obtained when the RH reached ≈ 90%, probably because of the destruction of the film morphology by the penetration of high concentration moisture. In comparison, the WOG strategy improved the reproducibility of OSCs across a wide range of RH: The WOG‐based devices achieved PCEs of >18.0% across RH from 20 to 80% and reached an average PCE of 18.5% at ≈ 90% RH, which we ascribed to the suppressed water‐induced trap and maintained film morphology as discussed below. The relatively narrow PCE distribution and the sustained high performance under large variations in ambient RH suggested good reproducibility in the device fabrication and highlight the WOG for direct processing in the ambient atmosphere. Given that the storage stability in the open‐air is essential for the fabrication of large‐scale OSCs, especially before device encapsulation, hence, we fabricated the PM6:L8‐BO‐based OSCs without and with WOG to further investigate the potential of this strategy. The controlled PM6:L8‐BO device stored at ≈ 40% RH suffered a severe drop of ≈ 70% in its initial PCE value after 800 h, while the device with WOG demonstrated notably enhanced stability under the same storage condition, maintaining 85% of its initial PCE after 800 h, which is intuitive revealed in the change of *J*‐*V* curves of the storage device (Figure 1e). Furthermore, the strong humidity endurance of the WOG‐based OSCs aroused our curiosity about its robustness under two extremely harsh conditions involving water.^[^ ^38^ ^]^ For the first one, the active layer was fabricated in air and then immersed in water for 10 min. The second, more harsh treatment involved in exposure to boiling water vapor for 10 min. As shown in the Figure 1f, the average PCEs of the immersed device and the device in boiling water vapor were 15.9% and 15.3%, extremely higher than those of the control ones (only 0.5%, Figure S4, Supporting Information), indicating the WOG strategy can remarkably inhibit the water‐induced degradation behavior. Despite differing from real‐world conditions, these experiments aid in predicting and enhancing the performance of devices in harsh environments. For instance, studying the behavior of photoactive films under water immersion or high RH is crucial for developing encapsulation techniques and stability strategies to protect solar cells from environmental factors.^[^ ^45^, ^46^ ^]^

### 2.2 The Mechanism of the WOG

**Figure 2:**
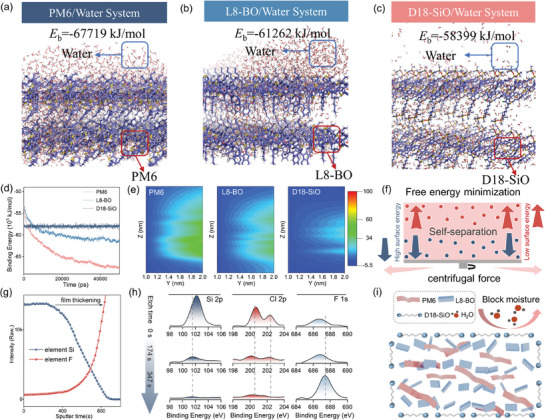
a–c) Simulated the interaction energy of PM6, D18‐SiO, and L8‐BO with water molecules. PM6, D18‐SiO, and L8‐BO were d) binding energy and e) water density distribution f) The schematic for the mechanism of D18‐SiO segregation during the film formation. g) TOF‐SIMS depth profiles of the Si^4+^, F^−1^, h) Depth etching profiles from the XPS analysis of the elements Si, Cl, and F in film, respectively. i) Schematic of the function of D18‐SiO in ternary film.

To evaluate the mechanism of WOG to protect against water erosion films, we conducted molecular dynamic simulation to mimic realistic model of the photovoltaic material/water system.^[^ ^47^, ^48^, ^49^, ^50^, ^51^ ^]^ The schematic representation of the optimized structures of PM6, L8‐BO and the guest polymer D18‐SiO that all contains four repeating D‐A units and 100 main backbone molecules for energetically favorable configurations were presented in Figures S5–S7 (Supporting Information). The obtained structures was further optimized under Compass force field for molecular structures and cell structures, and 2 × 6 × 10 cell expansion was carried out, with 10 000 water molecules added. Considering that the polymer chain is difficult to peristalsis because of the formation of stable thin film solid phase prior to the infiltration of water molecules, the polymer domain is regarded as a whole for dynamic simulation with the water. The detail dynamic optimization procedures were conducted (See simulation part in Supporting Information), followed by the determination of binding energy (*E*_b_) for the three interacting systems, and the partial scheme was displayed in **Figure** **2**a‐c. The greater the absolute value, the stronger the interaction, and vice versa.

As shown in the Figure 2d, *E*_b_ for the most stable states of Water (Wat)/D18‐SiO system were estimated to be ‐58399 kJ mol^−1^, lower than those of Wat/PM6 system (−67719 kJ mol^−1^) and Wat/L8‐BO systems (−61262 kJ mol^−1^), indicating that the D18‐SiO exhibit weaker interaction compared to PM6 and L8‐BO. The interaction variation trend can be also verified in the center‐of‐mass radical distribution functions curves of the PM6, D18‐SiO and L8‐BO cluster relative to the water molecules that weakest *g*(r) and can be obtained among the three clusters (Figure S8, Supporting Information). This demonstrated the stable optimized structure found for water D18‐SiO complex, with molecular water in a stable maximum intermolecular distance. The water densities across the entire composition range were computed through the dynamic simulation of the three different systems and compared with the experimental data, as illustrated in Figure 2e. The density of water within the same range of D18‐SiO clusters exhibit a significant decrease in comparison with those of the other two clusters. These results indicate that WOG weakens the interaction with water molecules through hydrophobic characteristics of siloxane terminated side chains of polymer, mitigating the moisture condensation on the blend film surface and inhibiting water penetration. The dramatically reduced density of water within active layers and around the surface of the film via the designed water‐obstructing guest should be an important factor that realize high stability in open‐air, and even in high humidity environment.

**Figure 3:**
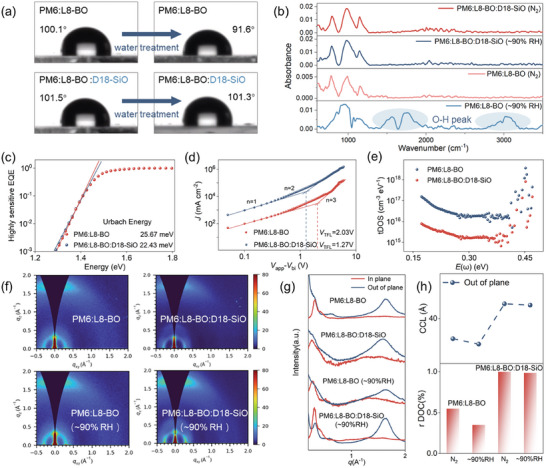
a) Contact Angle image of blend films against ambient moisture. b) FTIR spectra of PM6:L8‐BO film and PM6: L8‐BO: D18‐SiO film in different environment. c) s‐EQE of two systems at absorption onset and calculated Urbach energies; d) *J‐V* characteristics of hole‐only devices based on PM6:L8‐BO and PM6: L8‐BO: D18‐SiO (n represents the slope of the fitting line. e) trap density of states spectra of PM6:L8‐BO and PM6: L8‐BO: D18‐SiO based devices. f) 2D‐GIWAXS patterns of the optimal blended films. g) The in‐plane (IP) and out‐of‐plane (OOP) extracted line‐cut profiles of the corresponding blended films. h) Comparison of four blends CCL and rDOC.

Subsequently, we further studied the distribution of the guest D18‐SiO in the blend film to explore the mechanism of WOG.^[^ ^52^ ^]^ During the spin coating of active layer solution, the centrifugal force provides a strong driving force for the self‐assembly behavior of materials.^[^ ^53^, ^54^ ^]^ The component with high‐surface‐energy tends to migrate toward the substrate to minimize the free energy of the system. Conversely, the guest with hydrophobic features tends to aggregate on the film surface (Figure 2f). Time‐of‐flight secondary ion mass spectrometry (TOF‐SIMS) analysis (Figure 2g) indicated that D18‐SiO predominantly accumulates near the upper surface of the membrane, while PM6 and L8‐BO are more abundantly distributed in the deeper regions of the film. The self‐assembly behavior of D18‐SiO is also verified by using X‐ray photoelectron spectroscopy (XPS).^[^ ^55^ ^]^ As shown in Figure 2h, before etching, the prominent peaks at 102 eV corresponding to the silicon element and the peaks at 200 and 202 eV corresponding to the chlorine element indicates that D18‐SiO is mainly located on the surface of the active layer. As the etching depth increases, the peak at 687 eV corresponding to PM6 and L8‐BO components, exhibits an obviously upward trend, which is consistent with the results obtained from TOF‐SIMS. This suggests the siloxane terminated side chains with hydrophobic characteristics is mainly located near film surface. Such distribution properties were also be revealed by exploring the impact of WOG on the surface energy via contact angle measurement (**Figure** **3**a).^[^ ^56^ ^]^ For the binary film, the measured contact angles decreased from 100.1° to 91.6° after 5 min of immersion in water. However, the contact angles remained nearly constant when the binary film treated with WOG. To investigate the potential interference of PEDOT: PSS on the self‐migration of the siloxane coating due to its hygroscopic properties, we tested the contact angle of WOG devices with or without PEDOT: PSS after RH ≈ 90% air treatment, and calculated the surface tension. As shown in the Table S5 (Supporting Information), the slightly difference in surface tension between the two devices under 90% RH conditions indicates the negligible interference of the PEDOT: PSS on the self‐migration of the siloxane coating. These results further corroborate that WOG can induce a robust hydrophobic active layer, thereby reducing water condensation on the surface of the blend film. Since the D18‐SiO with siloxane terminated side chains exhibit weak intermolecular interaction with water in comparison with PM6 or L8‐BO, the hydrophobic polymer forms a soft framework around the photovoltaic materials, and act as a capping layer to block moisture penetration.

### 2.3 Trap Density Suppression with WOG

Previous research has shown that robust interactions between water molecules and the active layers can induce the formation of traps, which should be a significant factor affecting carrier transport and extraction, and ultimately resulting in rapid attenuation of device performance.^[^ ^15^, ^16^, ^17^, ^18^, ^19^, ^20^ ^]^ To explore the effect of WOG on traps within film during long‐term storage in air (≈ 90% RH), we perform Fourier transform infrared (FTIR) spectroscopy. As shown in Figure 3b, the FTIR spectrum of control film prepared in air and stored for 24 h exhibits additional peaks compared to the film treated in N_2_ environment. Specifically, there is a stretching vibration absorption peak of free water O─H at 3400 cm^−1^, and an O─H stretching vibration peak at 1640 cm^−1^, indicative of water molecules associated with photovoltaic materials via hydrogen bonding, which can conduct to the increase in the trap density and continuous degradation of the active layers. In contrast, almost unchanged FTIR spectrum can be observed for the WOG film processed in corresponding two different conditions, indicative of the weakening of the interaction between water molecules and active layer, as well as the reduction of the water induced trap density.

To further elucidate the efficiency of WOG in inhibiting the trap formation to a certain degree,^[^ ^57^, ^58^, ^59^, ^60^ ^]^ high‐ sensitive external quantum efficiency (s‐EQE) was conducted to investigate its impact on energetic disorder. By fitting the vicinity of the bandgap of s‐EQE spectrum, the air‐processed (≈ 90% RH) WOG device displays a lower Urbach energy (22.4 meV) than the control one (25.7 meV) (Figure 3c), indicative of a lower degree of energy disorder within WOG devices. To provide a more comprehensive characterization of the disorder within the blend film, the total trap density of the air‐processed devices with and without WOG were quantitatively measured using the space‐charge‐limited‐current (SCLC) method (Figure 3d). The total trap density in the device can be calculated according to the following Equation (1):

(1) $$V_{\mathrm{TFL}}=\mathrm{qNt}d^{2}/2\varepsilon_{0}\varepsilon$$

where *V*_TFL_ is the trap‐filled limit voltage, *q* is the elementary charge, *N*_t_ is the total trap density, *d* is the active layer thickness, *ε*_0_ is the permittivity of vacuum, and *ε* is the relative dielectric constant. The total trap density in the device with WOG was calculated to be 4.21 × 10^16 ^cm^−3^, which is lower than that in the controlled one (6.73 × 10^16 ^cm^−3^). Thus, the WOG can efficiently inhibit the localization of charge carriers, improving the charge‐carrier mobility and suppressing charge recombination in the devices at open‐air. Furthermore, we quantitatively measured trap density in these devices processed at high humidity (≈ 90% RH) by using the thermal admittance spectroscopy (TAS) method. According to the TAS results (Figure 3e), the WOG device exhibits a lower trap density of states (tDOS) of 1.49–7.61 × 10^15^ cm^−3^ eV^−1^ than the control device (0.16–1.75 × 10^16^ cm^−3^ eV^−1^) within the energy depth of 0.15–0.4 eV, while WOG device has similar tDOS values with controlled one within the energy depth of 0.40–0.45 eV. It indicates that WOG can efficiently reduce trap density of devices at high humidity, which is quite consistent with the results from SCLC method. The charge‐carrier mobilities of PM6:L8‐BO‐based devices were determined using the SCLC method. As expected, the electron and hole mobilities of the WOG devices at 90% RH were 4.84 × 10^−4^ and 5.03 × 10^−4^ cm^2^ V^−1^ s^−1^, respectively, and almost one order of magnitude higher than control devices (4.33 × 10^−5^ cm^2^ V^−1^ s^−1^ for electrons and 3.91 × 10^−5^ cm^2^ V^−1^ s^−1^ for holes) (Figure S15, Supporting Information). The higher charge‐carrier mobilities of WOG device at high humidity condition echo the lower trap density within the blend film. The above results indicate that WOG strategy can inhibit the formation of water‐induced trap density, thereby maintaining excellent exciton dissociation and charge transport properties for air‐processed devices, which may be related to the evolution of the blend film morphology with WOG strategy.

Subsequently, the effect of the WOG strategy on the crystallization and stacking of blend films were investigated by grazing‐incidence wide‐angle X‐ray scattering (GIWAXS) measurements.^[^ ^61^, ^62^ ^]^ The 2D GIWAXS images and 1D cut‐off curves of blend films in the air and N_2_ atmosphere were displayed in Figure 3f,g. The PM6:L8‐BO films processed in N_2_ without WOG treatment displayed the preferred face‐on orientation with a clear π–π stacking peaks at *q* ≈ 1.63 Å in the OOP direction and a lamellar peak at *q* ≈ 0.29 Å in the IP direction, which is consistent with the previous reports.^[^ ^5^, ^63^ ^]^ Similar peaks, with dramatically decreased intensity and reduced crystalline coherent length (CCL) for π‐π stacking, can be observed in air‐processed (≈ 90% RH) films without WOG treatment, which demonstrates the decreased crystallinity and decayed film possibly due to the water‐induced trap. For the two blend films with WOG treatment, it was found that both films processed under different condition maintained the face‐on orientation, indicating the WOG does not change the molecular orientation property. Noticeably, the calculated (010) CCL of films processed in N_2_ and air (≈ 90% RH) were estimated to be 20.93 Å, showing similar π‐π stacking structure. In addition, the relative degree of crystallinity (rDOC) for the PM6:L8‐BO under different processing condition was calculated based on the method reported in previous studies (Figure 3h). The calculated rDOC of lamellar stacking (100) were 0.88 and 0.42 in the films processed in N_2_ and open air without WOG, while the rDOC for the WOG films processed in N_2_ and open air increased to 1.0 and 0.95, meanwhile the d‐spacing of (010) packing decreased (Table S6, Supporting Information), indicating the ordering of packing was enhanced in the film and defects can be minimally generated with WOG treatment. Thus, utilizing WOG strategy by introducing of D18‐SiO as the guest can facilitate the molecular stacking and sustain strong crystallinity for the film under high humidity condition. This result can be explained by the fact that D18‐SiO is solidified to form a framework around at surface and act as capping layer to extrude PM6 and L8‐BO to a certain extent to favor a more tightly packed film and inhibit the formation of trap density. The enhanced crystallinity of WOG film is in excellent agreement with the improved hole mobility and better air stability as discussed above.

### 2.4 Carrier Dynamics Analysis

**Figure 4:**
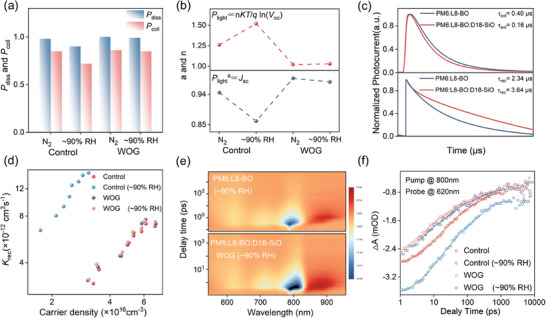
a) *P*_diss_ and *P*_coll_ of control and WOG devices. b) Dependences of *J*_SC_ and *V*_OC_ on *P*_light_. c) TPC and TPV of control and WOG devices. d) Bimolecular recombination rate constants (*k*_rec_) versus carrier density of control and WOG devices. e) 2D TA profile at different time delays of the control and WOG devices. f) Comparison of the hole‐transfer kinetics for the corresponding blends.

The improved *J*_SC_ and FF are the main factors that contribute to the enhanced PCE of WOG OSCs under high humidity. To better understand the exciton dissociation (*ɳ*_diss_) and charge collection (*ɳ*_coll_) mechanism in the active layers, the dependency of photocurrent (*J*_ph_) versus effective voltage (*V*_eff_) was plotted for the optimal device without and with WOG treatment, as shown in **Figure** **4**a. Apparently, *ɳ*_diss_ and *ɳ*_coll_ values of the devices were seriously affected by the processing condition. The PM6:L8‐BO device fabricated in air (≈ 90% RH) without WOG showed a distinctly decreased *P*_diss_ (88.3%) and *P*_coll_ (70.3%) compared with those cast in the N_2_ glovebox (*P*_diss_ = 96.5% and *P*_coll_ = 83.5%). In contrast, the WOG devices have less reduction in both *P*_diss_ and *P*_coll_ values in high humidity air processing (*P*_diss_ = 96.7% and *P*_coll_ = 83.0%) compared with that in N_2_ processing (*P*_diss_ = 97.4% and *P*_coll_ = 84.5%), indicative of sufficient exciton diffusion to donor/acceptor interfaces for dissociation and maintained charge collection properties, benefitting from the low trap density in high humidity air processing, well explain the synchronous enhancement of *J*_SC_ and FF in these devices.

In addition, the slope of *nkT*/*q* in the function of *V*_OC_/*nkT* = *q*ln*P* (*q* is elementary charge, *k* is Boltzmann constant, and *T* is temperature) is an indicator of the trap‐assisted recombination (geminated recombination), which could be characterized by the *V*_OC_ ‐*P*_light_ intensity relationship, as shown in Figure 4b. The control PM6:L8‐BO device processed in open air (≈ 90% RH) displayed a significantly increased slope (1.52 *kT/q*) compared with that cast in the N_2_ glovebox. For WOG devices, the comparable fitted slope between the device in high humidity air processing and in N_2_ atmosphere indicates that WOG strategy can remarkably suppress the severe trap recombination when processed in high humidity air. The bimolecular recombination was further characterized by the *J*_SC_ ‐*P*_light_ intensity relationship, as expressed by equation *J*_SC_∝*P*_light_^a^, in which the linear fitting slopes *α* values are similar when processed in high humidity air (0.97) and in N_2_ atmosphere (0.98) via WOG treatment. However, smaller *α* values were obtained for the controlled device in the corresponding two processing condition (0.86 and 0.94), indicating that bimolecular recombination was limited for WOG devices processed in high humidity air.

Subsequently, transient photocurrent (TPC) and transient photovoltage (TPV) measurements were performed to examine the influence of WOG on charge carrier dynamics within devices processed in ≈ 90% RH. The charge extraction time (τ_ext_) and recombination times (τ_rec_) of the photocarriers were determined by fitting the TPC decays under short‐circuit conditions and the TPV decays under open‐circuit conditions, respectively. As illustrated in Figure 4c, the charge extraction times for WOG devices was 0.16 µs, markedly shorter than the 0.40 µs obtained for the controlled device. These results indicate that WOG facilitates more efficient charge extraction inside device processed in high humidity air, thereby enhancing EQE response and improving FF value. Simultaneously, in comparison to the control device (2.34 µs), the prolonged τ_rec_ time (3.64 µs) observed for the device with WOG suggests a greatly suppressed recombination loss in these OSCs, which may be ascribed to high charge‐carrier mobility in WOG devices for its low trap density.

The integration of TPV and charge extraction (CE) measurements were investigated to quantify the bimolecular recombination loss inside devices in high humidity air (≈ 90% RH).^[^ ^64^, ^65^ ^]^ The analysis of carrier density (n) values of the WOG‐based devices inferred from CE at an open circuit under different light intensities was higher than the control one over the entire light intensity range, reflecting the more efficient exciton dissociation in the WOG devices. Then, the effectiveness of WOG was confirmed by estimating the bimolecular recombination rate constants (*k*_rec_) extracted from the carrier lifetime and carrier density,^[^ ^66^ ^]^ according to the formula: *k*_rec_ = 1/(𝜆+1) nτ, where 𝜆 is the recombination order from Figure S23 (Supporting Information). As illustrated in Figure 4d, the *k*_rec_ values of the WOG devices processed in high humidity air exhibited a consistent decrease compared to the controlled one across all *V*_OC_ ranges, aligning with the reduction in bimolecular recombination and the enhancement of FF and *J*_SC_ values in WOG devices.

To further trace the effect of WOG on the exciton dissociation kinetics of the blend film in high humidity air and N_2_ atmosphere processing, the femtosecond transient absorption (fs‐TA) spectra were measured.^[^ ^67^, ^68^, ^69^ ^]^ An 800 nm excitation wavelength was used to selectively excite L8‐BO. The color plots and corresponding spectra with varying decay time of the four blend films are depicted in Figure 4e and Figure S26 (Supporting Information). Similar spectra can be observed for these films that with the decay of the acceptor ground state bleach (GSB) peak at 700 nm ≈ 800 nm, the GSB peak of donor appears at 620 nm and increases in the first 50 ps, which can be assigned to hole transfer from L8‐BO to donor. The hole‐transfer rates are fitted using biexponential function, where the ultrafast τ_1_ represents the exciton dissociation time at the D/A interfaces and the slower τ_2_ represents the exciton diffusion time toward the interface before dissociation (Figure 4f). The PM6:L8‐BO processed without WOG in high humidity air (≈ 90% RH) showed a significant increased τ_1_ and τ_2_ values (τ_1_ = 27.1 ps and τ_2_ = 30.0 ps) compared with that as cast in the N_2_ glovebox (τ_1_ = 5.4 ps and τ_2_ = 6.4 ps), indicating that the water‐induced trap may limit the rapid exciton diffusion and dissociation at D/A interface. However, the device using WOG strategy significantly alleviates this process, as evidenced by the comparable τ_1_ /τ_2_ values for the film when processed in the N_2_ glovebox or air conditions with ≈ 90% RH. This suggests that WOG strategy could facilitate exciton diffusion to D/A interface, while mitigating the reduction in exciton dissociation rate in high humidity condition, thereby achieving highly efficient and stable OSCs.

### 2.5 OSCs Fabrication with WOG by Blade Coating Application and the Universality

Encouraged by the inspiring results using WOG strategy in inhibiting water‐induced trap density and maintaining outstanding performance of OSCs prepared in high humidity condition, we further employed blade‐coating technique to fabricate 1 cm^2^ OSCs in open‐air, which is significant for the deployment of large‐scale manufacturing. In this study, PM6:L8‐BO with WOG was deposited in open‐air (≈ 40% RH) using air‐knife assisted blade‐coating technique, from non‐halogenated o‐xylene (XY). The high boiling point of XY (140 °C) will reinforce the aggregation, so utilizing laminar air‐knife quenching together with hot‐casting substrate at a temperature of 50 °C could effectively control the drying kinetics of polymer wet film. The PCE of the champion WOG‐based device improved from 15.1% to 17.3%, with the *J*_SC_ rising from 24.5 mA cm^−2^ to 26.1 mA cm^−2^ and the FF from 72.5% to 76.2%, relative to the controlled device. The *V*_OC_ and *J*_SC_ dependence on illumination intensity suggested suppressed trap‐assist recombination and reduced bimolecular recombination in WOG‐based device. The *J*_SC_ calculated from the EQE measurements matched closely with the *J*_SC_ obtained under AM1.5G illumination (24.8 mA cm^−2^ vs 22.7 mA cm^−2^).

**Figure 5:**
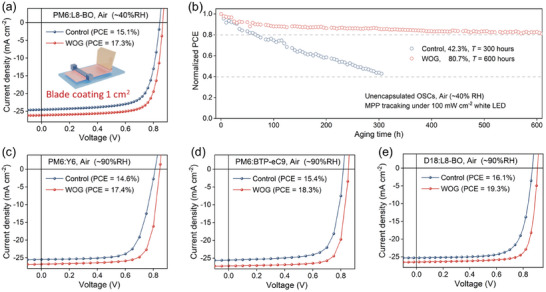
a) *J*‐*V* curves for the blade‐coated control and WOG devices at a 1.0 cm^2^ active area. b) Maximum power point (MPP) stability test of the control and WOG devices under 1‐sun equivalent illumination from white LEDs under MPP conditions in 40% RH. *J‐*‐*V* curves for the control device and WOG devices with different active layer systems: c) PM6:Y6, d) PM6: BTP‐eC9, e) D18:L8‐BO.

Stability tests for the both inverted and conventional OSCs under different conditions were carried out to investigate the contributions of WOG to device stability. First, as shown in Figure S27 (Supporting Information), the PCE of inverted device with WOG retains 86% of its initial value after being heated at 85 °C for 360 h, which is superior to inverted device without WOG (78%). However, for conventional devices, it can be found a dramatically discrepancy on thermal stability with and without WOG. The WOG device demonstrated robust performance with ≈ 20% decrease after 192 h thermal aging at 85 °C. In contrast, the devices without WOG displayed decreased thermal stability, retaining 54% of their initial efficiencies after 72 h under same thermal aging condition. This indicates that WOG strategy has greater potential application value for conventional OSCs. Second, the unencapsulated conventional devices was assessed under continuous 1‐sun‐equivalent white LED illumination at MPP in air (**Figure** **5**b, the specific spectrum for LED illumination is provided in Figure S29, Supporting Information). The devices with WOG maintained 80.7% of the initial PCE in open air with the RH of ≈ 40% after 600 h ‐benefiting from the surface hydrophobicity capping introduced by the accumulation of the D18‐SiO, compared with 42.3% of the initial PCE after 300 h for the controlled devices. In addition, the maintained blend morphology and suppressed water‐induced trap density, aided by WOG treatment, could also be significant factor leading to improved device stability.

At last, the universal nature of the WOG is demonstrated in other three photovoltaic systems, including donor D18, and three different Y‐series acceptors, Y6, L8‐BO, BTP‐eC9. As presented in Figure 5c,d, the photovoltaic blends with WOG exhibit higher PCE than the ones without WOG which processed in high‐humidity ambient conditions (≈ 90%RH). Meanwhile, different from the controlled device with low reproducibility under high humidity condition, the WOG improved repeatability of OSCs due to the obtained narrow performance distributions. For D18:L8‐BO: D18‐SiO blends (Figure 5e), the PCE is ≈ 1.3 times higher than the D18:L8‐BO blends, with narrow average PCE of 18.5 ± 0.7%, validating the applicability of the WOG strategy. Overall, the above results highlighted the significant potential of the WOG strategy for sustaining the excellent performance and high stability of OSCs under high humidity condition, which is promising for large‐scale and low‐cost manufacturing.

## 3 Conclusion

Despite the remarkable rise in the efficiency of OSCs, the water‐induced instability and inferior reproductivity and performance is widely identified as a critical hurdle for upcoming commercialization. This study developed a WOG strategy using a siloxane decoration based‐polymer D18‐SiO as a capping layer, which attenuates the interaction of the active layer with water molecules and minimizes moisture penetration, because of hydrophobic and low surface free energy characteristics of WOG, while preserving nano‐micromorphology with high crystallinity and tight packing properties. Benefiting from the reduced defects, suppressed molecular recombination and promoted exciton dissociation with the WOG strategy under high humidity conditions, the PCE of >19% was achieved for spin coating OSCs fabricated across an RH range of 20 to 90%, and concurrently, blade coating 1 cm^2^ OSCs demonstrated a PCE of >17% at 90% RH. Moreover, the unencapsulated device retained 80.7% of its initial performance in air (≈ 40% RH) after 600 h maximum power point tracking under continuous light illumination. We believe that the WOG strategy adopted here show the great potential toward the commercial production of OSCs.

## Conflict of Interest

The authors declare no conflict of interest.

## Supporting information



Supporting Information

## Data Availability

The data that support the findings of this study are available from the corresponding author upon reasonable request.
