# Supplementary material for: Siloxane Decorated Water‐Obstructing Guest for Efficient Air‐Processed OSCs
Source: Adv Sci (Weinh). 2025 Feb 20;12(15):2412190. doi: 10.1002/advs.202412190 (PMC12005760; doi:10.1002/advs.202412190)
Supplement: Supplementary file 1 — Supporting Information [file ADVS-12-2412190-s001.docx]

**Supporting Information for**

**Siloxane Decorated Water-Obstructing Guest for Efficient Air-processed OSCs**

Yurong He,^a^ Wentao Miao,^a^ Tianyu Hu,^a^ Junchi Su,^a^ Aziz Saparbaev,^b^ Ming Wan,^a^ Jingnan Wu,^c^ Yuda Li,^d^ Huimin Xiang,^a^ Ergang Wang,^c^ Xunchang Wang,*^a^ Renqiang Yang*^a^

Y. He, W. Miao, T. Hu, J. Su, Dr. M. Wan, Dr. H. Xiang, Dr. X. Wang, Prof. R. Yang

Key Laboratory of Optoelectronic Chemical Materials and Devices (Ministry of Education), School of Optoelectronic Materials & Technology, Jianghan University, Wuhan 430056, China

E-mail: wangxc@jhun.edu.cn,

Dr. A. Saparbaev
Institute of Ion-plasma and Laser Technologies, National University of Uzbekistan, Tashkent 100174, Uzbekistan

J. Wu, Prof. E. Wang

Department of Chemistry and Chemical Engineering, Chalmers University of Technology, Göteborg 41296, Sweden

Dr. Y. Li

Key Laboratory of Novel Biomass-based Environmental and Energy Materials in Petroleum and Chemical Industry, School of Chemical Engineering and Pharmacy, Wuhan Institute of Technology, Wuhan 430205, China.

**Materials and Synthesis.**

All the reagents, unless otherwise specified, were purchased from Sigma-Aldrich Co., *J&K*, and Tokyo Chemical Industry Co., Ltd., and were used without further purification. PM6, L8-BO, D18, Y6, BTP-ec9 was purchased from Solarmer Materials Inc. All air and water-sensitive reactions were carried out under N_2_. The general synthetic routes of D18-SiO were shown in Scheme S1. The detailed synthetic procedures are as follows.


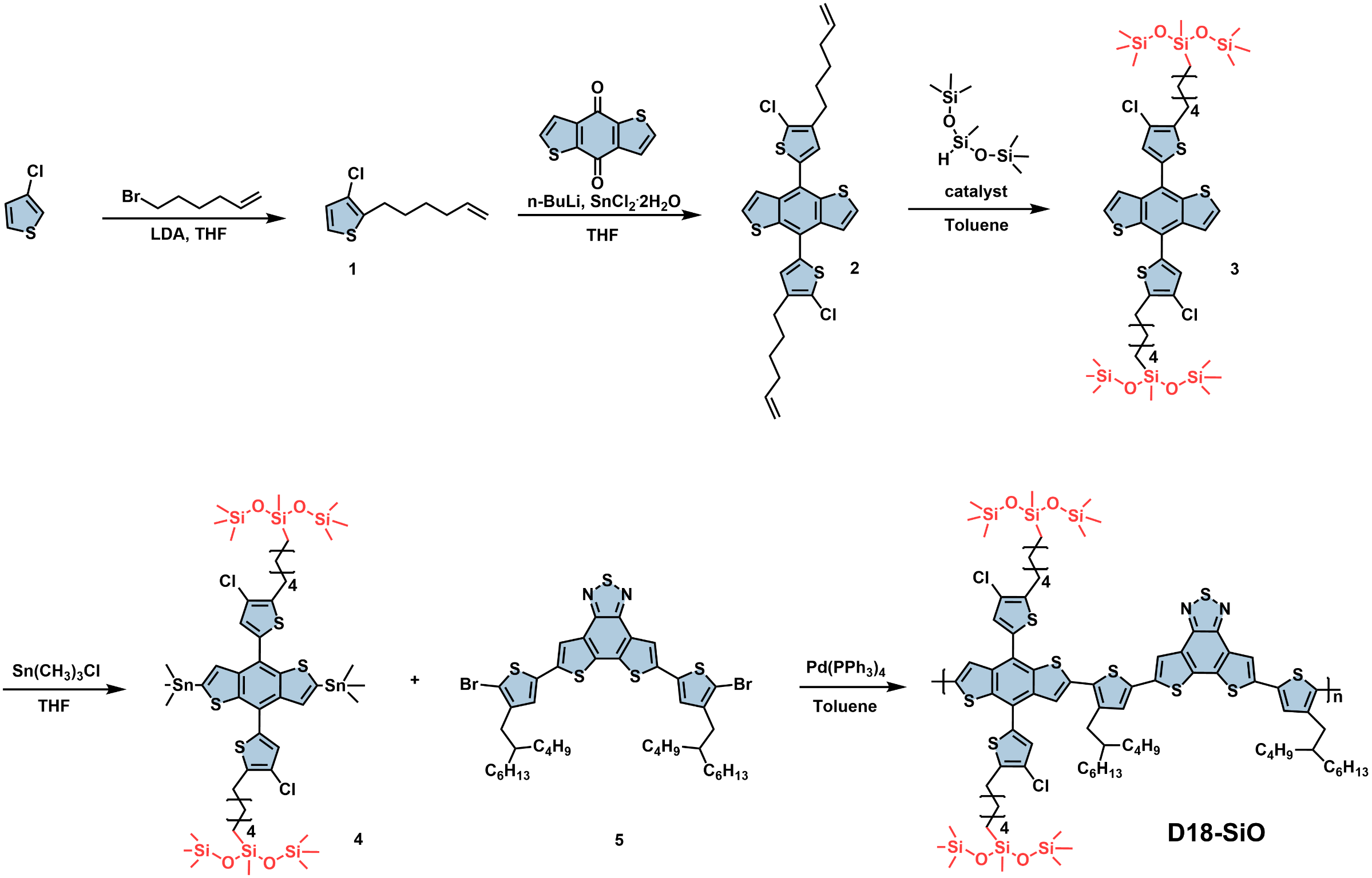


**Scheme S1.** The synthetic route of D18-SiO.

**Synthesis of Polymer D18-SiO** ^1-3^

**Compound 1:** Under an inert gas atmosphere, diisopropylamine (30.72 g, 303.6 mmol) and 150 mL of anhydrous and anaerobic tetrahydrofuran solvent. *n*-BuLi (126.5 mL, 303.6 mmol) dissolved in *n*-hexane solution was added at -40 °C. After addition, it reacts at -40 °C for 1 h, and then transfers to 25 °C at room temperature for another 1 h. 3-chlorothiophene (30 g, 253 mmol) and 150 mL of anhydrous and anaerobic tetrahydrofuran solvent in an inert gas atmosphere. The LDA reagent prepared above is added to the mixture at -78 °C and reacts at -78 °C for 2 h after addition. 6-bromo-1-hexene (49.5 g, 303.6 mmol) was added to the above reaction solution and reacted at -78 °C for 1 h, then transferred to 25 °C at room temperature for another 16 h. Thereafter, distilled water was added thereto to terminate the reaction. After extraction with methylene chloride, water was removed from the extract with sodium sulfate (Na_2_SO_4_) and the extract free of water was subjected to a separation process using silica gel column chromatography to obtain compound 1 as a colorless solid (52.4 g, 86%). ^1^H NMR (400 MHz, Chloroform-d): δ 7.08 (d, *J* = 5.3 Hz, 1H), 6.85 (d, *J* = 5.3 Hz, 1H), 5.81 (td, *J* = 16.9, 6.7 Hz, 1H), 5.06-4.91 (m, 2H), 2.83-2.75 (m, 2H), 2.09 (d, *J* = 7.5 Hz, 2H), 1.74-1.60 (m, 2H), 1.53-1.41 (m, 2H). ^13^C NMR (101 MHz, Chloroform-d): δ 138.59, 137.60, 127.49, 121.94, 121.85, 114.63, 33.42, 30.07, 28.24, 27.41.

**Compound 2:** We put the compound 1 (6.42 g, 32 mmol) and anhydrous THF (25 mL) in a two necked flask. Thereafter, the solution was cooled to 0 °C and *n*-BuLi (22 mL，34 mmol) was slowly added thereto. Next, we stirred the solution for 2 hours at a temperature of 50 °C. 4,8-dihydrobenzo[l,2-b:4,5-b] dithiophene-4,8-dione (2.2 g, 10 mmol) was added there to dropwise, and the mixture was stirred for 2 hours. After cooling the mixture to room temperature, add Tin (II) chloride dihydrate (19.42 g, 86.2 mmol) solution dissolved in 10% HCl (24 mL), and the mixture was stirred for 12 hours. Thereafter, distilled water was added thereto to terminate the reaction. After extraction with methylene chloride, water was removed from the extract with sodium sulfate (Na_2_SO_4_) and the extract free of water was subjected to a separation process using silica gel column chromatography to obtain compound 2 as a pale-yellow solid (2.35 g, 80%). ^1^H NMR (400 MHz, Chloroform-d): δ 7.54 (s, 2H), 7.15 (s, 2H), 5.85 (td, *J* = 16.9, 6.7 Hz, 2H), 5.10-4.97 (m, 4H), 2.95-2.84 (m, 4H), 2.15 (q, *J* = 7.0 Hz, 4H), 1.83-1.70 (m, 4H), 1.61-1.52 (m, 7H). ^13^C NMR (101 MHz, Chloroform-d): δ 140.22, 139.62, 138.47, 135.95, 134.47, 128.24, 125.64, 122.40, 121.58, 117.44, 114.81, 33.38, 29.95, 28.38, 27.76.

**Compound 3:** The dissolving compound 2 (2.35 g, 4 mmol) and 1,1,1,3,5,5,5-heptamethyltrisiloxane (1.89 g, 8.5 mmol) were dissolved in toluene (20 mL), and Karstedt’s catalyst 5 mg was added thereto. Thereafter, after stirring at 110 °C for 12 hours. Thereafter, distilled water was added thereto to terminate the reaction. After extraction with methylene chloride, water was removed from the extract with sodium sulfate (Na_2_SO_4_) and the extract free of water was subjected to a separation process using silica gel column chromatography to obtain 2.89 g(Yield:70%) of a pale-yellow solid material compound 3. Compound 3 was sensitive and used directly in the next step.

**Compound 4:** Compound 3 (2.06 g, 2 mmol) was dissolved in anhydrous THF and the solution was cooled to -78 °C in a nitrogen atmosphere, and *n*-BuLi (3.2 mL, 5 mmol) was added thereto. After stirring at -78 °C for 30 minutes, trimethyltin chloride (7 mL,7 mmol) was quickly added thereto. After stirring at room temperature for 12 hours, After the reaction is complete, the deionized water quenching reaction is added. It was extracted three times with deionized water and n-hexane solution. After the extraction is completed, the obtained phases are washed 5 times with deionized water to remove excess toxic tin reagents. (Organotin reagents should be treated with saturated potassium fluoride, and then subsequent waste liquid treatment). The resulting organic phase is dried with anhydrous sodium sulfate. After the solvent is removed by the rotary evaporator, the oil obtained is recrystallized. First, the oil is washed with ethanol solution to remove impurities. Then recrystallized with n-hexane and ethanol to obtain compound 4 as a yellowish powder solid tin salt (1.98 g, yield:73%). ^1^H NMR (400 MHz, Chloroform-d): δ 7.63 (s, 2H), 7.25 (s, 2H), 2.94-2.87 (m, 3H), 1.77 (dt, *J* = 15.2, 7.6 Hz, 5H), 1.54-1.26 (m, 6H), 0.72-0.05 (m, 21H). ^13^C NMR (101 MHz, Chloroform-d): δ 141.40, 141.31, 136.92, 135.39, 134.46, 128.69, 125.97, 119.89, 119.62, 31.28, 31.02, 28.63, 28.55, 27.16, 27.08, 26.09, 24.55, 23.44, 21.17, 15.74, 14.27, 11.92.

**D18-SiO:** The compound 4 (136 mmg, 0.1 mmol) and compound 5 (90.7 mg, 0.1 mmol) with siloxane side chain were weighed and added with 5 mL of toluene solvent. Purge in nitrogen atmosphere for 30 min. Then the catalyst tris (dibenzylideneacetone)dipalladium Pd (PPh_3_)_4_ (5 mg) was added, and then purged under nitrogen atmosphere for 1 h. Reflux was heated at 110° C for 16 h. The resulting polymer is dripped into a methanol solution and dried to obtain a solid dark polymer. The polymerization is purified by Soxhlet extraction (with methanol, dichloromethane, and chloroform), and the excess unreactive monomer compounds and catalysts are washed away, while the smaller molecular weight polymers are removed. Concentrate the resulting solid to remove excess solvent. Finally, the polymer was recovered from the chloroform fraction to a solid by methanol precipitation and dried under vacuum to obtain crude product D18-SiO.

**Instruments and characterizations**

**Nuclear magnetic resonance (NMR) spectroscopy**.

^1^H and ^13^C NMR spectra were measured on a Bruker AV-500 MHz spectrometer in deuterated solvents at room temperature. Chemical shifts were recorded with tetramethylsilane (TMS) as the internal reference.

**Absorption spectra measurements.**

UV-vis absorption spectra were carried out via Hitachi U-4100 spectrophotometer. spectrophotometer.

**Cyclic Voltammetry.**

Cyclic voltammetry (CV) measurements were conducted at a scan rate of 0.1 V s ^−1^ with a three-electrode cell system in a nitrogen bubbled 0.1 M solution of tetra-*n*-butylammonium hexafluorophosphate (*n*-Bu_4_NPF_6_) in acetonitrile at room temperature. The working, reference, and counter electrodes for CV measurement were based on glassy carbon, Ag/Ag^+^(Ag/AgNO_3_) solution in acetonitrile, and platinum wire, respectively. The Ag/Ag^+^ reference electrode was calibrated using an internal standard of a ferrocene/ferrocenium redox pair with an oxidation potential of -4.8 eV with respect to a zero-vacuum level. Accordingly, the HOMO/LUMO energy levels were calculated using the equations HOMO/LUMO (eV) = − (*E*_(ox/red)_ ^onset^ − *E*_(ferrocene)_^onset^ + 4.8).

**Solar Cell Device Fabrication and Characterization.**

Normalized devices: The OSCs were fabricated with a device configuration of ITO/PEDOT: PSS/ active layer/PDINN/Ag. The ITO-coated glass substrates were sonicated successively with detergent, deionized water, acetone and isopropanol, and dried with nitrogen flow. Then, the ITO substrates were treated by ultraviolet-ozone for 15 min. The PEDOT: PSS solution was spin-coated onto ITO with 5000 rpm for 30 s as the hole transporting layer. Next, the ITO substrates with PEDOT: PSS were baked at 150 ℃ for 15 min. Typically, the ITO glasses were transferred into the N_2_-filled glove box. For the spin-coating devices, PM6: D18-SiO: L8-BO (WOG) is 1:0.03:1.2 in CF (7 mg mL^-1^ for donor) were then spin coated on the ITO/PEDOT: PSS layers. The optimized solvent additive is DIM, and the optimal content of DIM is 0.2 vol%. Then photoactive layers were treated with thermal annealing at 85 ℃ for 5 min. After cooling to room temperature, the PDINN solution in methanol with a concentration of 1 mg mL^-1^ was spin-coated on the top of photoactive layer at 3000 rpm for 30 s, giving a thin film of ~5 nm. Finally, 100 nm Ag electrode was deposited by thermal evaporation to complete the devices. The current-voltage (*J*-*V*) characteristics were measured with a Keithley 2450 source measurement unit. The OSCs were measured under an irradiation intensity of 100 mW cm^-2^ (AM 1.5G) by a Newport solar simulator. The EQE spectra were analyzed using an integrated system (LST-QE). The highly sensitive EQE was measured by using an integrated system (PECT-600, Enlitech), where the photocurrent was amplified and modulated by a lock-in instrument.

Inverted devices: The inverted OSCs were fabricated with a device structure of ITO/ZnO/active-layer/MoO_3_/Ag. The ZnO precursor was prepared by dissolving 1 g zinc acetate dihydrate and 280 µL ethanolamine in 20 mL of 2-methoxyethanol under stirring overnight for the hydrolysis reaction. The ZnO layer was formed by spin-coating the ZnO precursor solution (4000 rpm for 30 s) onto ITO substrates and then was annealed at 150 ℃ for 15 min under ambient conditions. The processing conditions of the active layers were the same as with the conventional OSCs. MoO_3_ (5 nm) and Ag (120 nm) layers were prepared by vacuum evaporation under vacuum condition of 5×10^-4^ Pa. A photon mask with a well-defined aperture size was used in the cell measurement to reduce the light piping and internal-scattering-induced edge effect to access *J*_SC_ more accurately. The solar-cell performance test used an Air Mass 1.5 Global (AM 1.5G) solar simulator (SS-F5-3A, Enlitech) with an irradiation intensity of 100 mW cm^-2^, which was measured by a calibrated silicon solar cell (SRC2020, Enlitech). The *J*-*V* curves were measured along the forward scan direction from -0.5 to 1 V, with a scan step of 50 mV and a dwell time of 10 ms, using a Keithley 2400 Source Measure Unit.

Water treated devices: For the extremely harsh treatments active layers, the PM6:L8-BO and PM6:D18-SiO: L8-BO solution was first spin-coated on surface of PEDOT: PSS. Then the surface of active layer was immersed with water for 10 min or the entire film was exposed in boiling water vapor for 10 min. After the harsh treatments, the surface water of the active layer was removed, and the active layer was annealed at 85°C for 5 minutes. Subsequently, a PDINN solution in methanol, with a concentration of 1 mg mL^-1^，was spin-coated onto the photoactive layer at 3000 rpm for 30 seconds, resulting in the formation of a thin film approximately 5 nm in thickness. Finally, a 100 nm silver electrode was deposited through thermal evaporation to complete the fabrication of the devices.

**Molecular Dynamic Analysis.**

All the small molecules and polymer units involved in this paper were firstly optimized by Gaussian software at B3LYP/6-31g** level and saved as mol2 format files ^4-6^. Then, sobtop software was used for cell expansion and GAFF2 force field parameters were obtained by DRIH method. In order to make the simulation results more accurate ^7-9^. The atomic charges involved in all force field parameters are replaced by RESP2 charges. The initial structure of the simulation system was generated by Packing Optimization for Molecular Dynamics Simulations (Packmol) program and periodic boxes were set ^10^. The molecular dynamics simulation was performed using the gromacs 2022.4 software package ^11^. The details are as follows:

1. System energy minimization

The 5000 step steepest descent method combined with 5000 step conjugate gradient method was used to optimize the system to avoid unreasonable contact.

2.NPT system pre-balance

The system was pre-balanced by NPT ensemble. V-rescale temperature coupling and parrinello-rahman pressure coupling were used to control the temperature of 298K, the pressure was maintained at 1 atm, the non-bonding truncation radius was 1.2 nm, and the integral step was 1fs.

3. Molecular dynamics simulation

After the system is balanced, switch to the Berendsen method. Bond length and bond Angle are constrained by LINCS algorithm. The two-way intercept is 1.2 nm, van der Waals interaction is used, and the long-distance electrostatic interaction is set by the particle-mesh Ewald method. The system saves a corresponding track file every 10.0 ps.

Related visualization and data analysis are carried out using the built-in scripts of VMD1.9.3, Multiwfn and gromacs.

**Contact Angle.**

The contact angle tests were performed on a contact angle meter (GBX DIGIDROP). The surface energy of the polymers was characterized and calculated by the contact angles of the two probe liquids using the Wu model (1):

Wu model:

$$\begin{aligned} \gamma_{water}\left( 1+cos\theta_{water} \right)=\frac{4\gamma_{water}^{d}\gamma^{d}}{\gamma_{water}^{d}{+\gamma}^{d}}+\frac{4\gamma_{water}^{p}\gamma^{p}}{\gamma_{water}^{p}{+\gamma}^{p}}\#\left( 1 \right) \end{aligned}$$

$$\gamma_{oil}\left( 1+cos\theta_{oil} \right)=\frac{4\gamma_{oil}^{d}\gamma^{d}}{\gamma_{oil}^{d}{+\gamma}^{d}}+\frac{4\gamma_{oil}^{p}\gamma^{p}}{\gamma_{oil}^{p}{+\gamma}^{p}}$$

$$\begin{aligned} \text{γ=}\text{γ}^{\text{d}}\text{+}\text{γ}^{\text{p}}\#\left( 2 \right) \end{aligned}$$

where γ is the total surface tension of acceptor and polymers; γ^d^ and γ^p^ are the dispersion and polar components of γ; γ^i^ is the total surface tension of the i material (i = water or diiodomethane); γ_i_^d^ and γ_i_^p^ are the dispersion and polar components of γ_i_; and θ_i_ is the droplet contact angle of the i material on the acceptor and polymers films.

**Fourier-transform infra-red (FTIR) measurements.**

The FTIR absorption spectra were performed on Infrared Spectrum Microscope (Nicolet is10 FT-IR spectrometer with diamond ATR crystals and a DTGS detector.). The measurement was based on a reflection mode.

**Time of Flight Secondary Ion Mass Spectrometry (TOF-SIMS).**

The TOF-SIMS were performed on TOF-SIMS 5 iontof. Experimental steps: lofting, vacuuming, sample testing. Analysis Parameters: PI(Bi1), Energy(30keV), Current(1.00pA), Area(100*100μm^2^), PIDD (7.49E+13 lons/cm^2^). The films of ITO/PEDOT: PSS/Active layer was prepared following the procedure that used to fabricate real devices.

**X-ray photoelectron spectroscopy (XPS).**

Sample Preparation: Spin film in argon glove box and allow all samples to dry. Then the sample transfer container is transferred to the XPS room. XPS signals are collected on the Thermo Fisher ESCALAB 250Xi scan XPS microprobe with Al Kα source.

**TAS measurement.**

Trap density of state (tDOS) was performed on Keysight 4980A and analyzed by using TAS method under angular frequency-dependent capacitance measurement (0.02 kHz to 2000 kHz). PM6:L8-BO and PM6: D18-SiO: L8-BO based devices for TAS measurement have the same device structure of ITO/PEDOT: PSS/Active layer/PDINN/Ag with the same processing conditions as their optimized OSCs in the work.

**Space-Charge-Limited Current Measurements.**

The hole and electron mobilities were calculated using the space charge limited current (SCLC) model with a device configuration of ITO/PEDOT: PSS/active layer/MoO_3_/Ag and ITO/ZnO/active layer/PDINN/Ag, respectively, where the current density is calculated by:

$$J= 9\varepsilon_{o}\varepsilon_{r}\mu V^{2}/(8L^{3})$$

where *J* stands for current density, $\varepsilon_{o}$ is the permittivity of free space, $\varepsilon_{r}$ is the relative dielectric constant of the transport medium, µ is the hole mobility, *V* is the voltage drop across the device (*V* = *V*_appl_ - *V*_bi_ - *V*_RS_, where *V*_appl_ is the applied voltage to the device, *V*_bi_ is the built-in voltage due to the difference in work function of the two electrodes, and *V*_RS_ is the voltage drop due to series resistance across the electrodes), and *L* is the thickness of the active layer.

**Grazing Incidence Wide-Angle X-Ray Scattering.**

2D-GIWAXS experiments were carried out on a GANESHA 300XL+ system from JJ X-ray. The instrument is equipped with a Pilatus 300K detector, with pixel size of 172 × 172 μm. The X-ray source is a Genix 3D Microfocus Sealed Tube X-Ray Cu-source with integrated Monochromator (30 W). The wavelength used is λ = 1.5418 Å. The detector moves in a vacuum chamber with sample-to-detector distance varied between 0.115 m and 1.47 m depending on the configuration used, as calibrated using silver behenate (d_001_ = 58.380 Å). The minimized background scattering plus high-performance detector allows for a detectable q-range varying from 3×10^−3^ to 3 Å^−1^ (0.2 to 210 nm). The sample was placed vertically on the goniometer and tilted to a glancing angle of 0.2° with respect to the incoming beam. A small beam was used to get a better resolution. The accumulation time was 30 minutes for each measurement. In-plane and out-of-plane line-cuts were obtained using SAXSGUI program.

**The Analysis of *J*_ph_ vs *V*_eff_ Relationships.**

The definition of *J*_ph_ is the difference between the current density under illumination (*J*_L_) and the dark current density (*J*_D_), with *V*_0_ denoting the voltage at which *J*_ph_ equals zero. Consequently, *V*_eff_ is defined as the difference between *V*_0_ and the applied voltage (*V*_appl_). When *V*_eff_ reaches a high value (> 4V), it is normally believed that generated excitons are fully collected, in which *J*_ph_ is equal to saturated current density (*J*_sat_). Then, we can calculate *J*_SC_/*J*_sat_ and *J*_max_/*J*_sat_ to describe exciton dissociation (*η*_diss_) and charge collection (*η*_coll_) efficiency. *J*_max_ is the *J*_ph_ at the maximal output point.

**Transient photocurrent (TPC), Transient Photovoltage (TPV) and Charge Extraction (CE) Measurements.**

TPC, TPV and CE measurements were obtained from a platform namely PAIOS which contains the characterization of Solar Cells. The TPV is based on monitoring the photovoltage decay upon a small optical perturbation during different constant bias light-intensity (using the same white LED for TPC measurements) and under open-circuit condition. Variable bias light intensities lead to a range of *V*_OC_ to be studied. A small optical perturbation (< 3% of the *V*_OC_, so that ∆*V*_OC_ ≪ *V*_OC_) is applied. The subsequent voltage decay is then also recorded to directly monitor nongeminate charge carrier recombination. The photovoltage decay kinetics of all devices follows a mono-exponential decay:$\delta V=Aexp(-t/\tau)$ where *t* is the time and $\tau$ is the charge carrier lifetime. The charge extraction (CE) technique was used to measure the charge carrier density n under open-circuit voltage condition. The device is illuminated and kept at open-circuit. After light is turned off, the voltage is set to zero or taken to short-circuit condition within a few hundreds of nanoseconds to extract the charges. To obtain the number of extracted charges, the current is integrated. Using the charge carrier lifetime obtained from TPV and charge carrier density from CE, the charge carrier lifetimes and charge carrier densities can be plotted. The charge carrier lifetime follows a power law relationship with charge density. The nongeminate recombination constant *k*_rec_ are then inferred from the carrier lifetimes and densities.

**Femtosecond Transient Absorption Spectroscopy Measurements.**

Femtosecond transient absorption spectroscopy measurements were performed on an Ultrafast Helios pump-probe system in collaboration with a regenerative amplified laser system from Coherent. An 800 nm pulse with a repetition rate of 1k Hz, a length of 100 fs, and an energy of 7 mJ pulse, was generated by a Ti: sapphire amplifier (Astrella, Coherent). Then the 800 nm pulse was separated into two parts by a beam splitter. One part was coupled into an optical parametric amplifier (TOPAS, Coherent) to generate the pump pulses at various wavelength. The other part was focused onto a sapphire plate and a YAG plate to generate white light supercontinuum as the probe beams with spectra covering 550-950 nm, respectively. The time delay between pump and probe was controlled by a motorized optical delay line with a maximum delay time of 8 ns. The pump pulse is chopped by a mechanical chopper with 500 Hz and then focused on to the mounted sample with probe beams. The probe beam was collimated and focused into a fiber-coupled multichannel spectrometer with CCD sensor. The energy of pump pulse was measured and calibrated by a power meter (PM400, Thorlabs). The samples used for TA measurements were obtained by spin-coating the neat and blend solutions on the quartz substrates.

Multi-exponential fitting method was used to fit the exciton lifetimes.
The used equations are shown as follows:

$$I_{(t)}\boldsymbol{=} A_{1}e^{-\frac{t}{\tau_{1}}}+ A_{2}e^{-\frac{t}{\tau_{2}}}+ A_{3}e^{-\frac{t}{\tau_{3}}}$$

$$\tau= A_{1}\times\tau_{1}+A_{2}\times\tau_{2}+A_{3}\times\tau_{3}$$

Where $A_{i}$ is the proportion of the corresponding lifetime of $\tau_{i}$.

**Open-air Blade Coating for Active Layers.**

The blade coat films were produced using Tol solutions with a PM6 concentration of 11 mg mL^-1^ for WOG blends at a temperature of 100 °C to ensure complete dissolution of materials. The films were deposited onto 50 °C ITO/PEDOT: PSS substrates at a speed of 20 mm s^-1^ in both forward and backward directions, with a blade-substrate gap of approximately 120 μm. Subsequently, the films were transferred to a nearby hotplate and annealed at 100 °C for 1 minute. The aforementioned procedures were conducted under ambient atmospheric conditions with RH of 40%. The N_2_ knife was employed to facilitate the drying of the film and mitigate any potential morphological damage resulting from excessively slow evaporation. This tool is operated by a gas pipeline that releases nitrogen with adjustable intensity and direction, typically parallel to the film surface.

We implemented the following strategies to maintain the wet state of the photoactive film during the coating process: 1) Substrate preheating: In our experiments, the substrate was preheated to 50 °C before coating. which not only promoted the evaporation of solvents, but also helped to maintain the appropriate wetness of the film layer during the coating process. This preheating step facilitated the evaporation of solvents and contributed to maintaining the desired level of wetness in the film layer throughout the coating process. 2) Nitrogen knife drying technique: We employed the nitrogen knife (N_2_-knife) technique for rapid drying. This technique effectively regulates the evaporation rate of the solvent on the surface of the film and prevents over-drying of the film before back-coating. 3) Precise control of the boiling point of the solvent: Given that the boiling point of the solvent is about 110 °C, we strictly monitor the temperature during the coating process to avoid exceeding the boiling point of the solvent, thus preventing the film from drying too quickly.

**
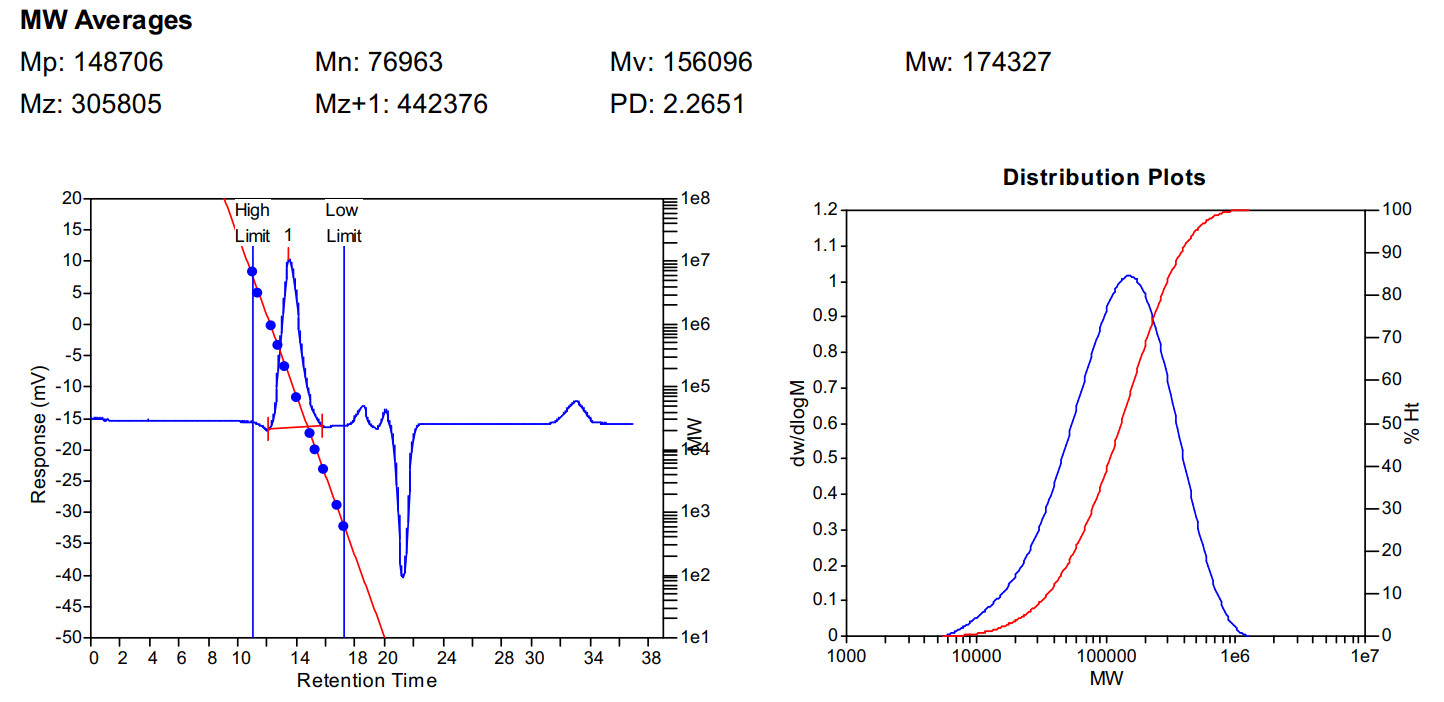
**

**Figure S1.** The number-average molecular weight (*M*_n_) and polydispersity index (Ð).


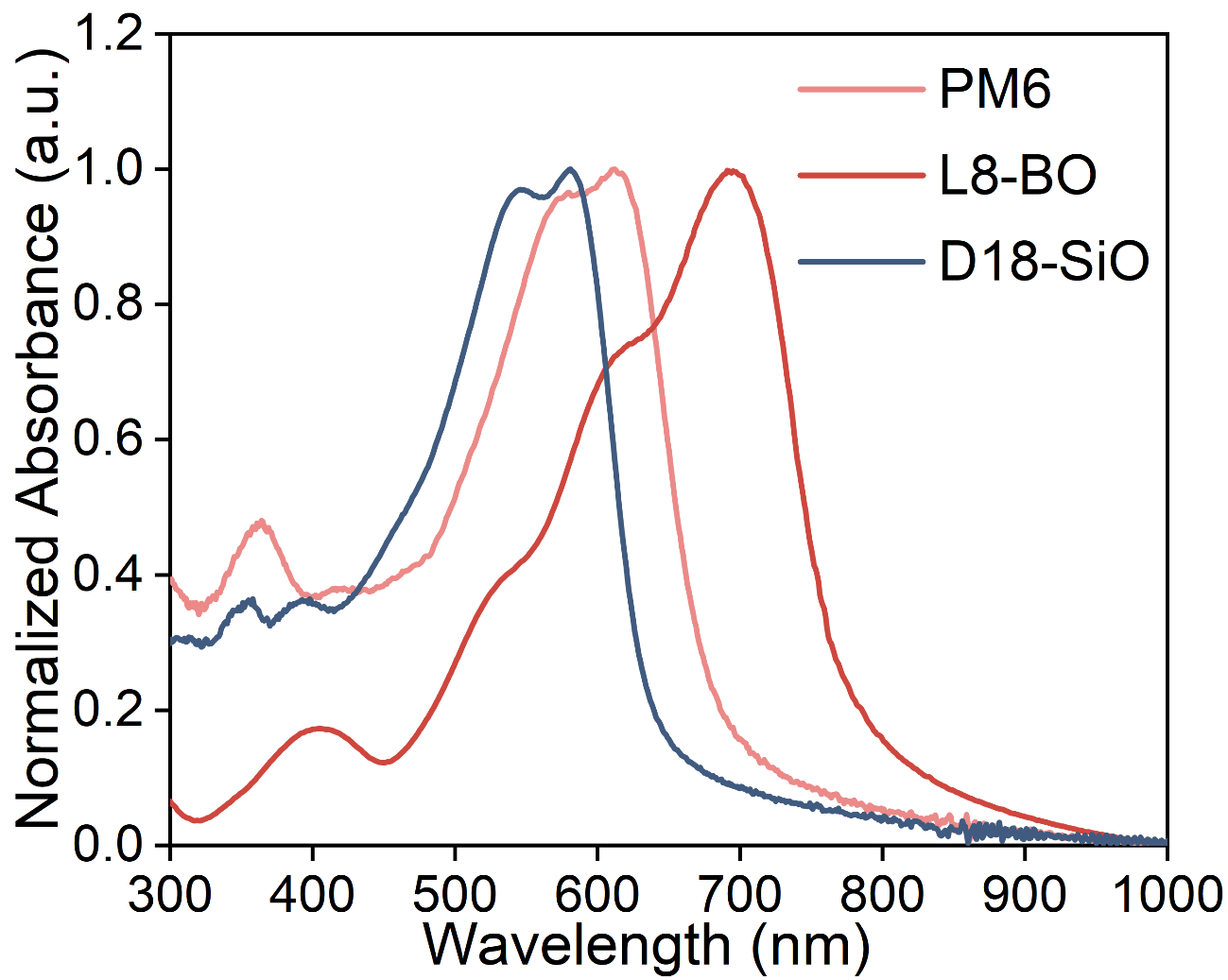


**Figure S2.** UV-vis spectra of neat films of PM6, L8-BO and D18-SiO.


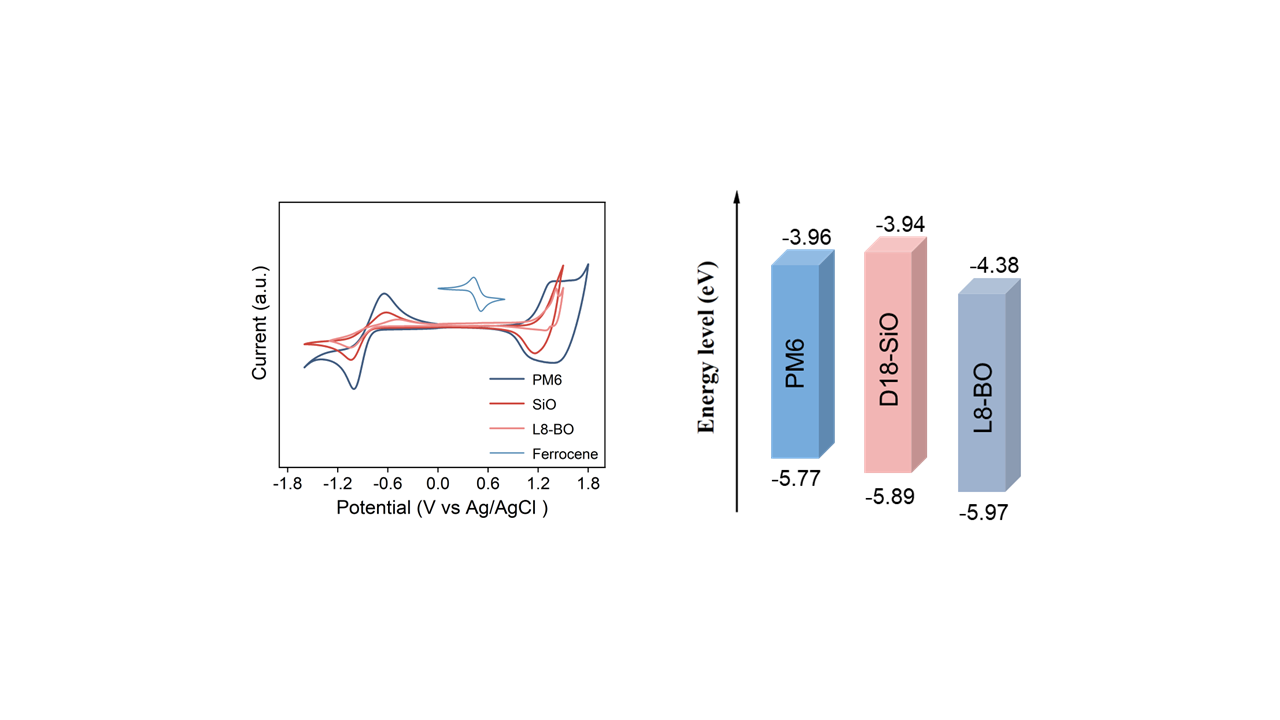


**Figure S3.** Cyclic voltammogram curves and Energy level diagrams of the donors and acceptor.

**
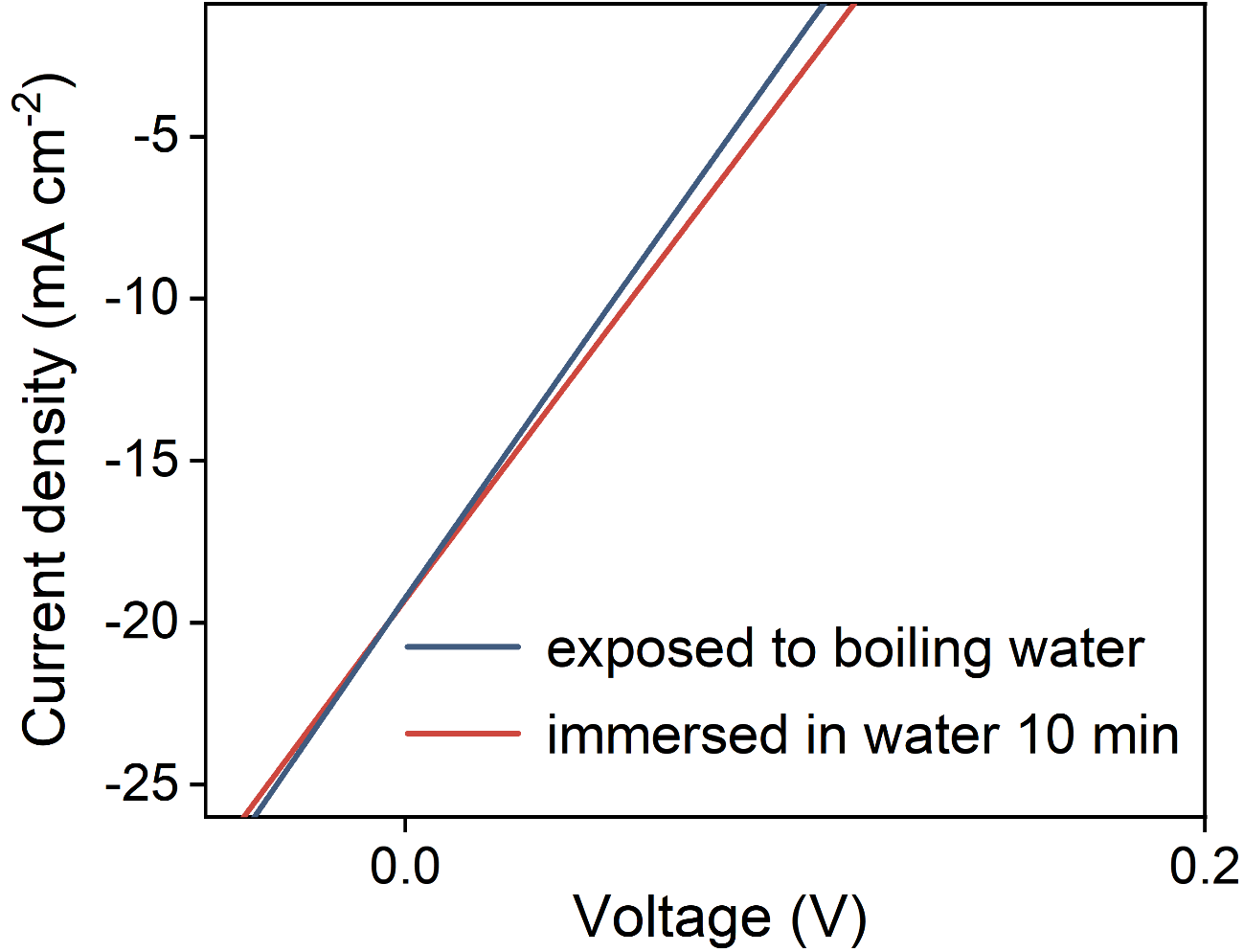
**

**Figure S4**. *J-V* curves of OSCs based on PM6:L8-BO binary active layer processed in air with different water environment.

**
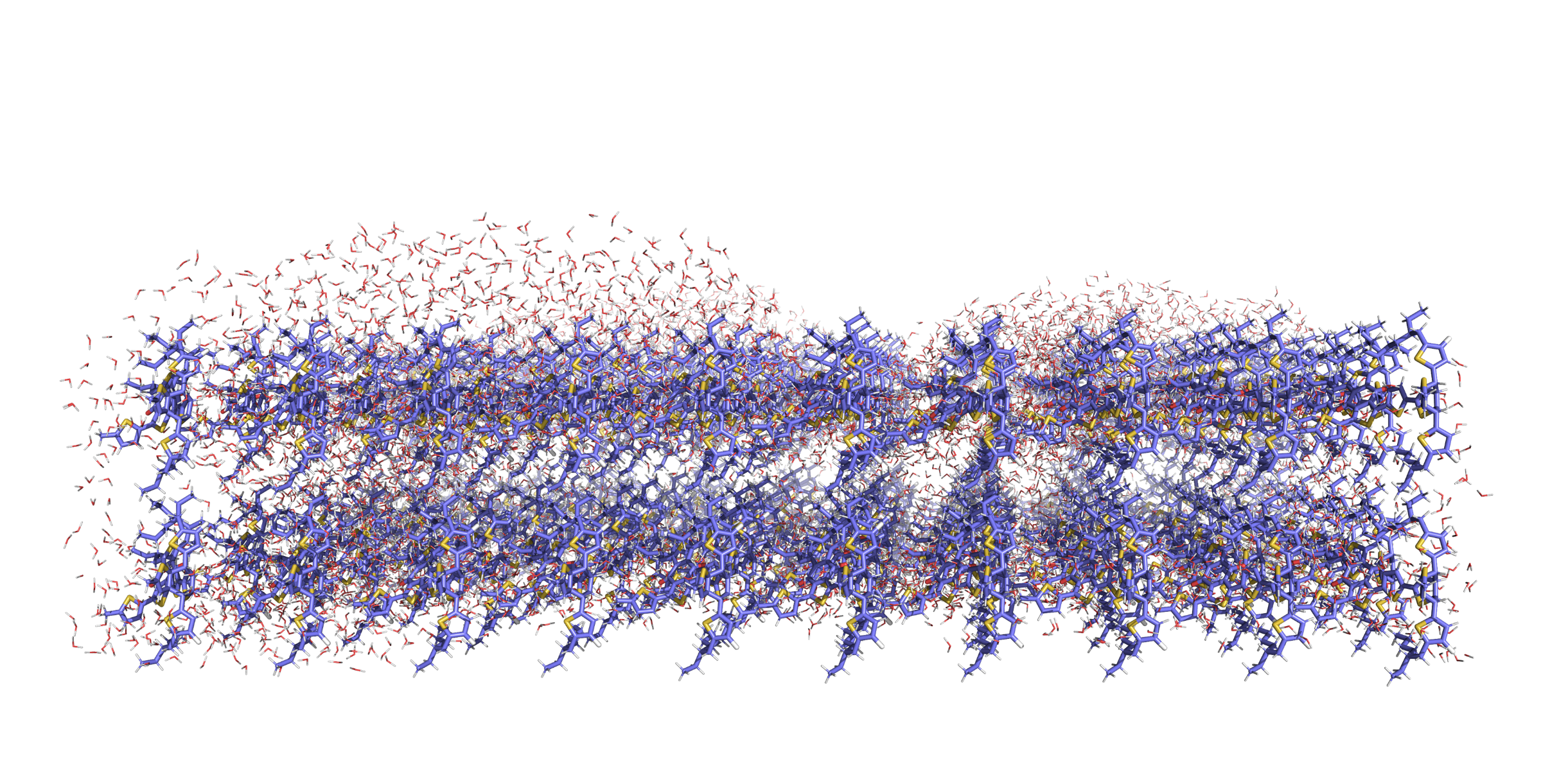
**

**Figure S5.** Schematic illustration of the interaction of PM6 molecules (blue) with water molecules (red).


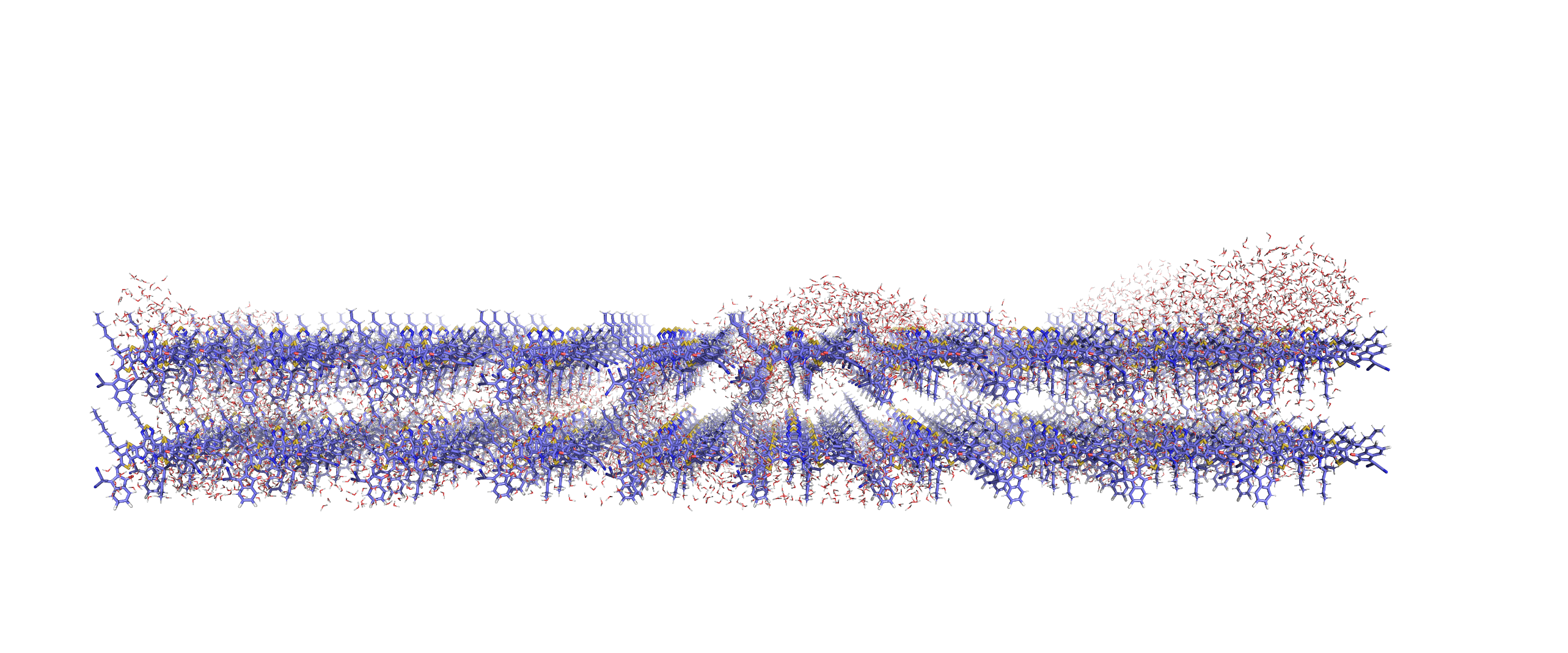


**Figure S6.** Schematic illustration of the interaction of L8-BO molecules (blue) with water molecules (red).


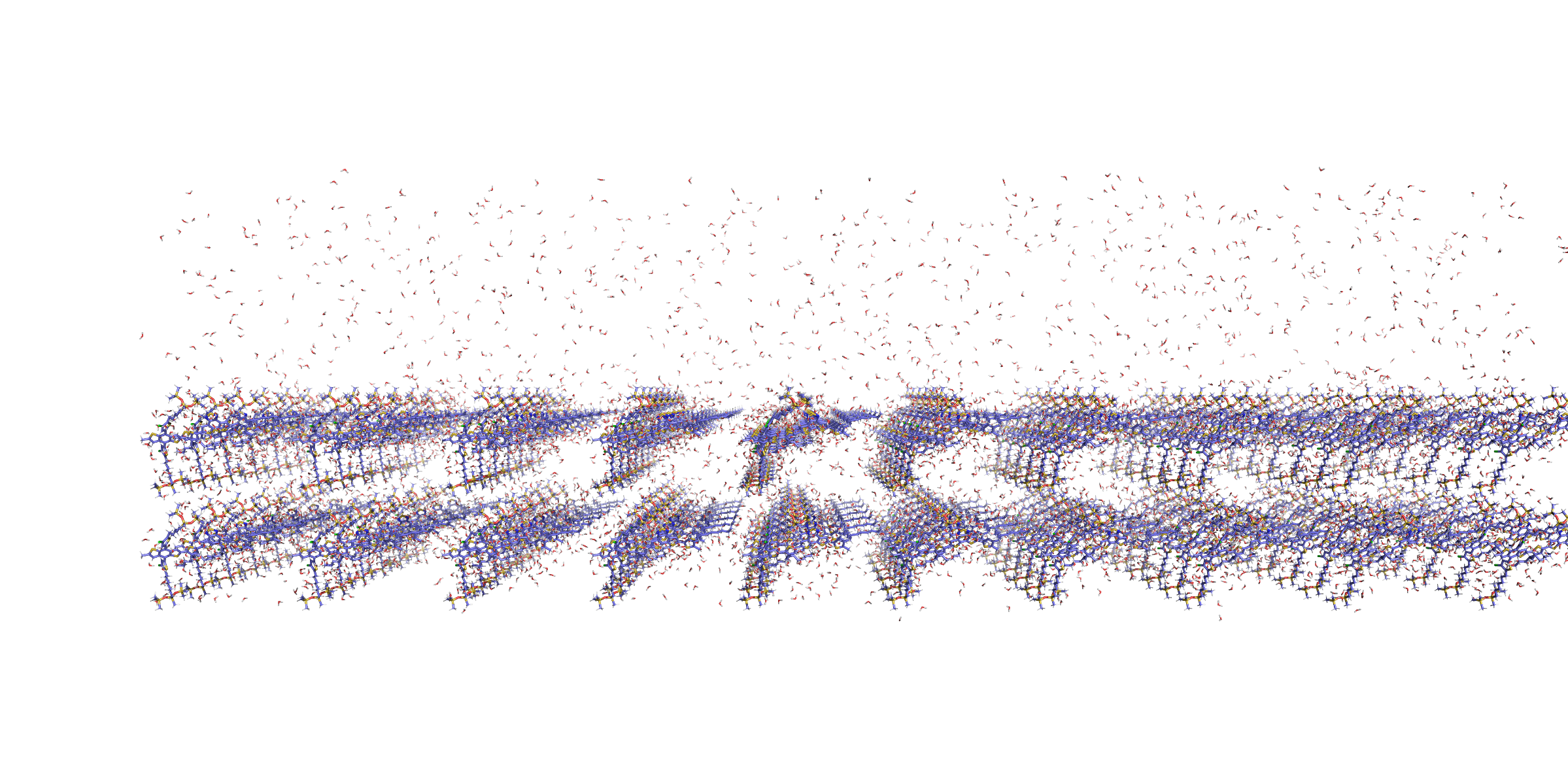


**Figure S7.** Schematic illustration of the interaction of D18-SiO molecules (blue) with water molecules (red).


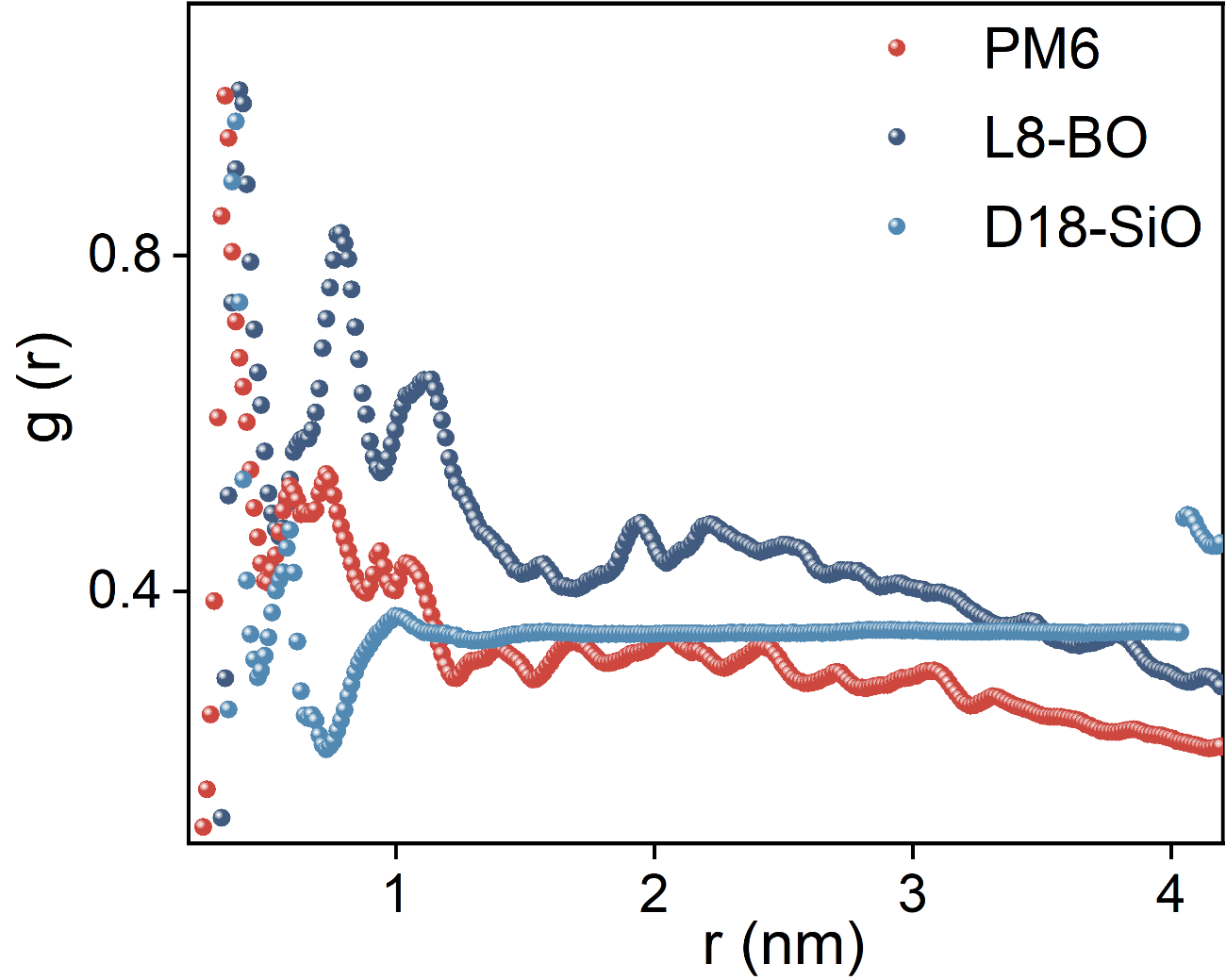


**Figure S8.** Center-of-mass radial distribution functions of the active layer materials and water molecule for the simulated films.


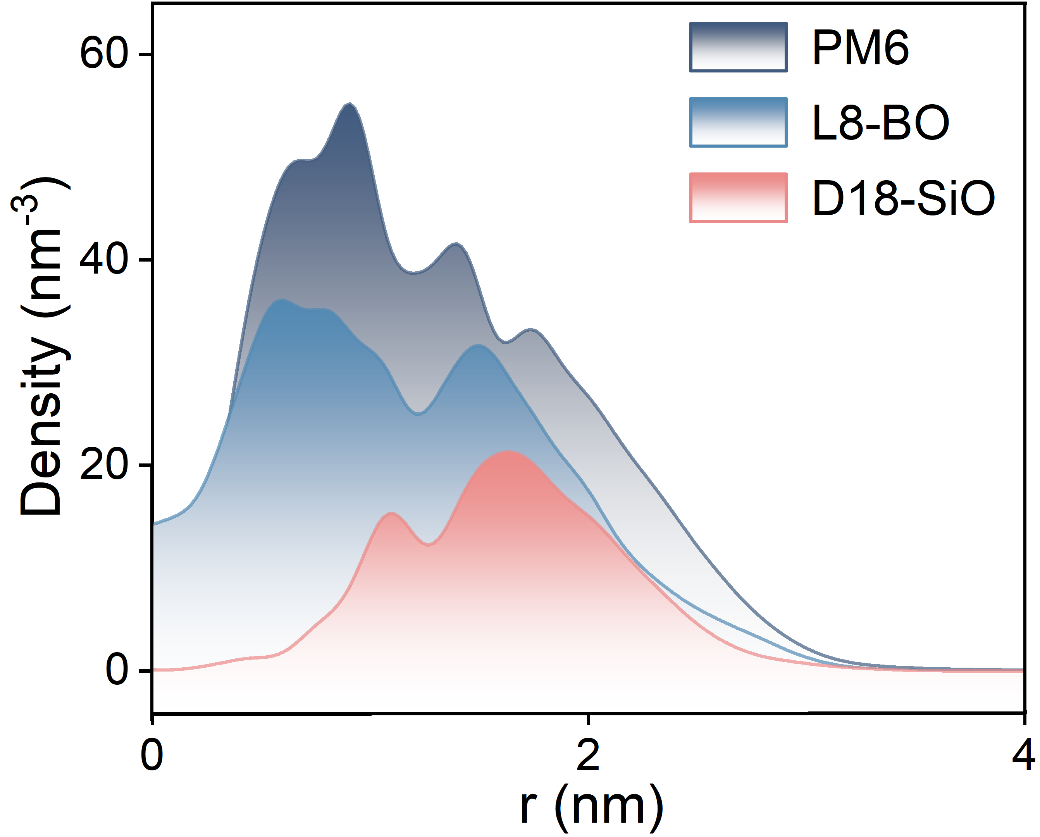


**Figure S9.** The water density distribution of the active layer material and the water molecule for the simulated films.


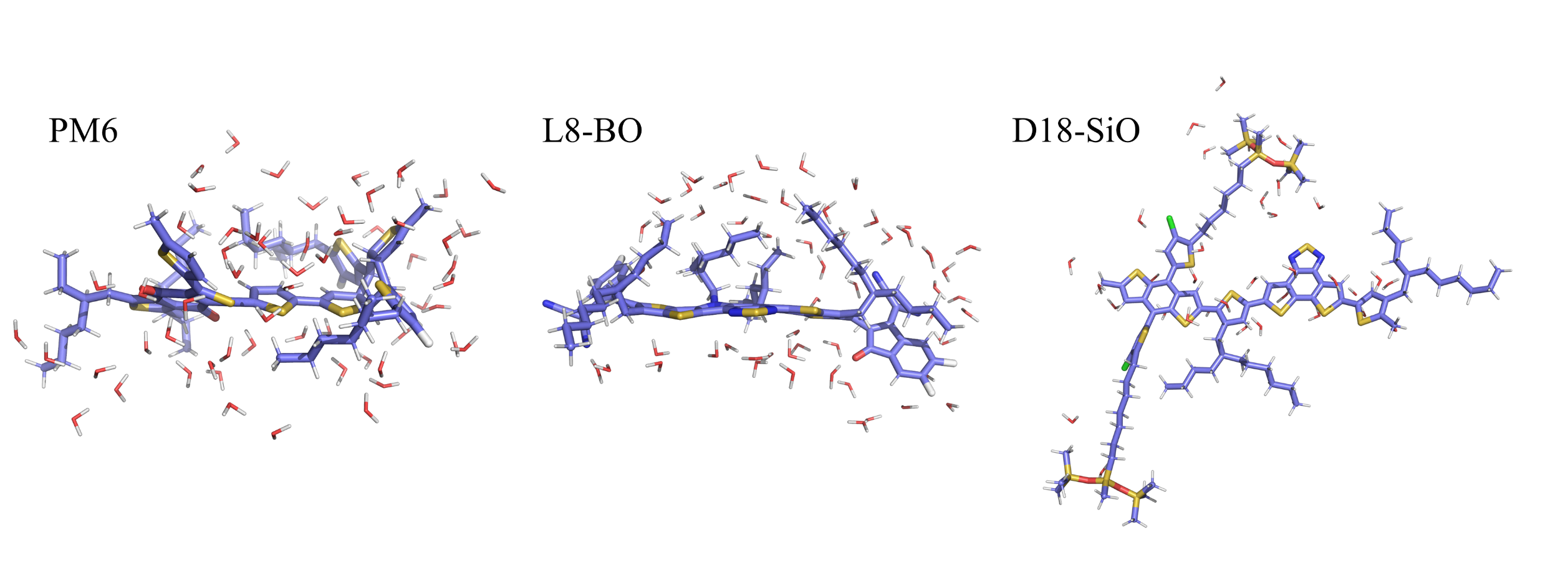


**Figure S10.** Detail view of the active layer material and the water molecule for the simulated films.

**
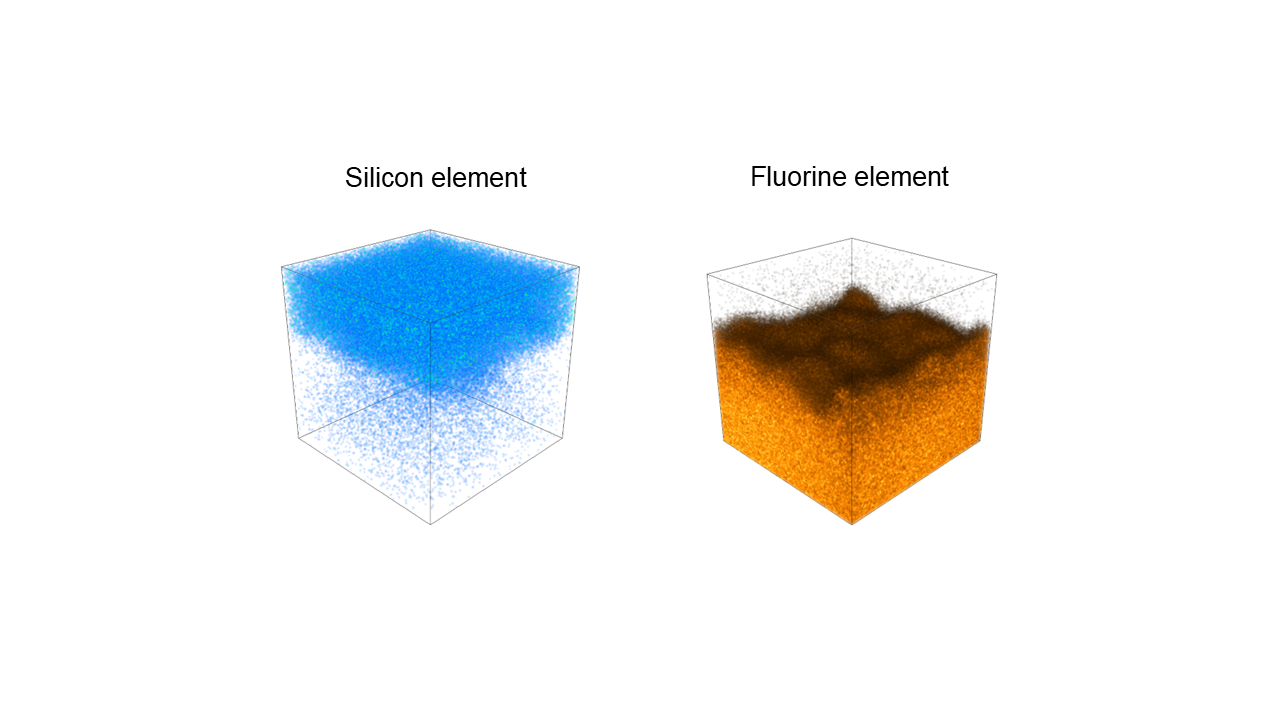
**

**Figure S11.** TOF-SIMS element distribution 3D model.


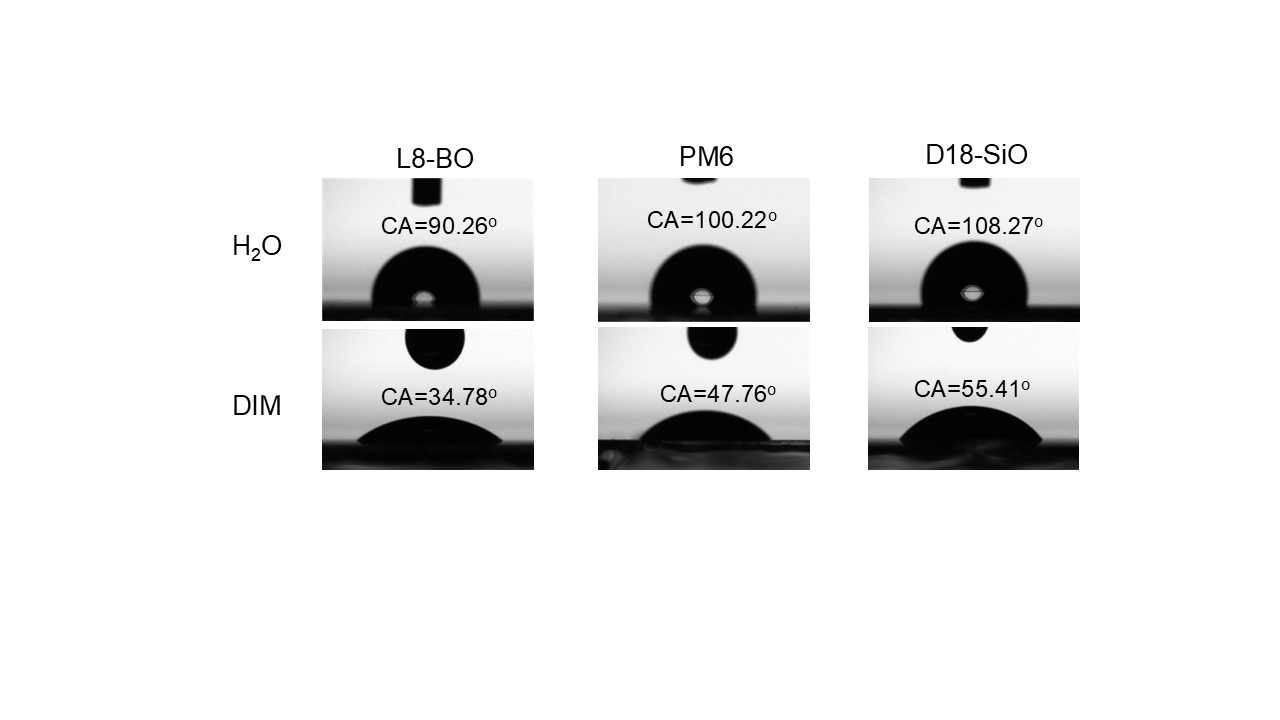


**Figure S12.** Contact angles of water and diiodomethane on polymer donors and L8-BO.


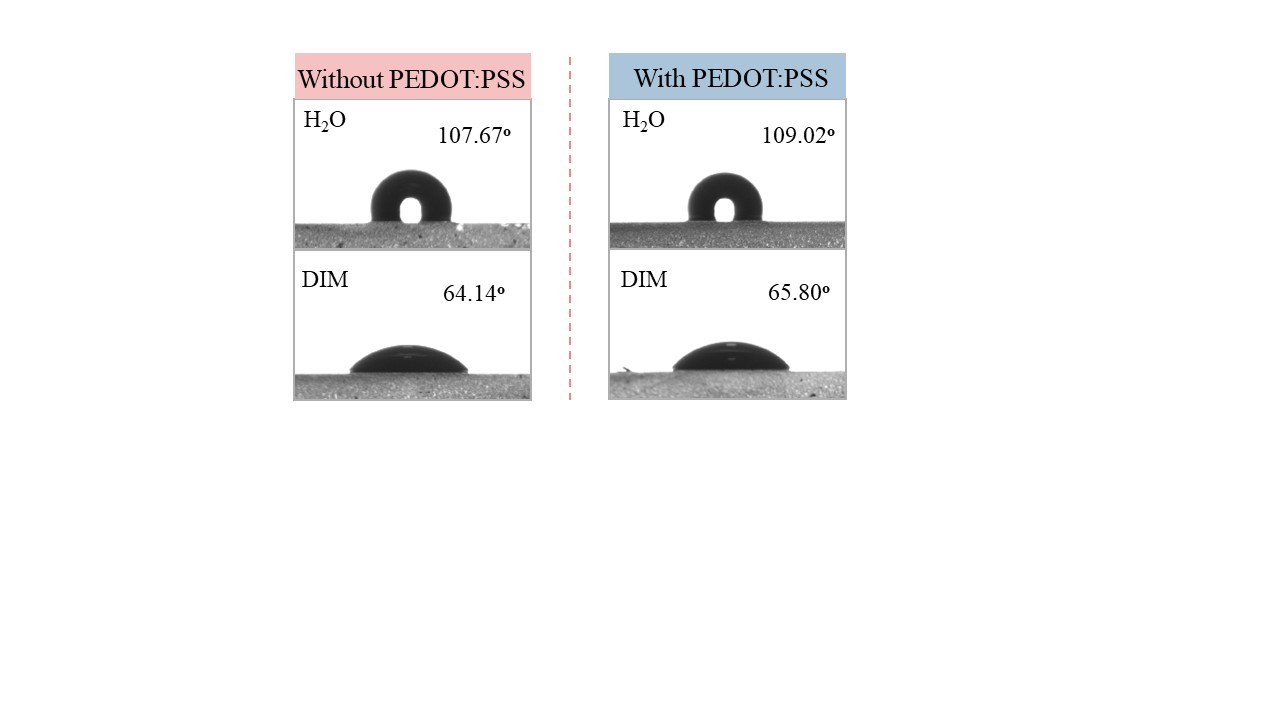


**Figure S13.** Contact angles of water and diiodomethane for WOG-based active layer treated at 90% RH without and with PEDOT: PSS as the HTL.


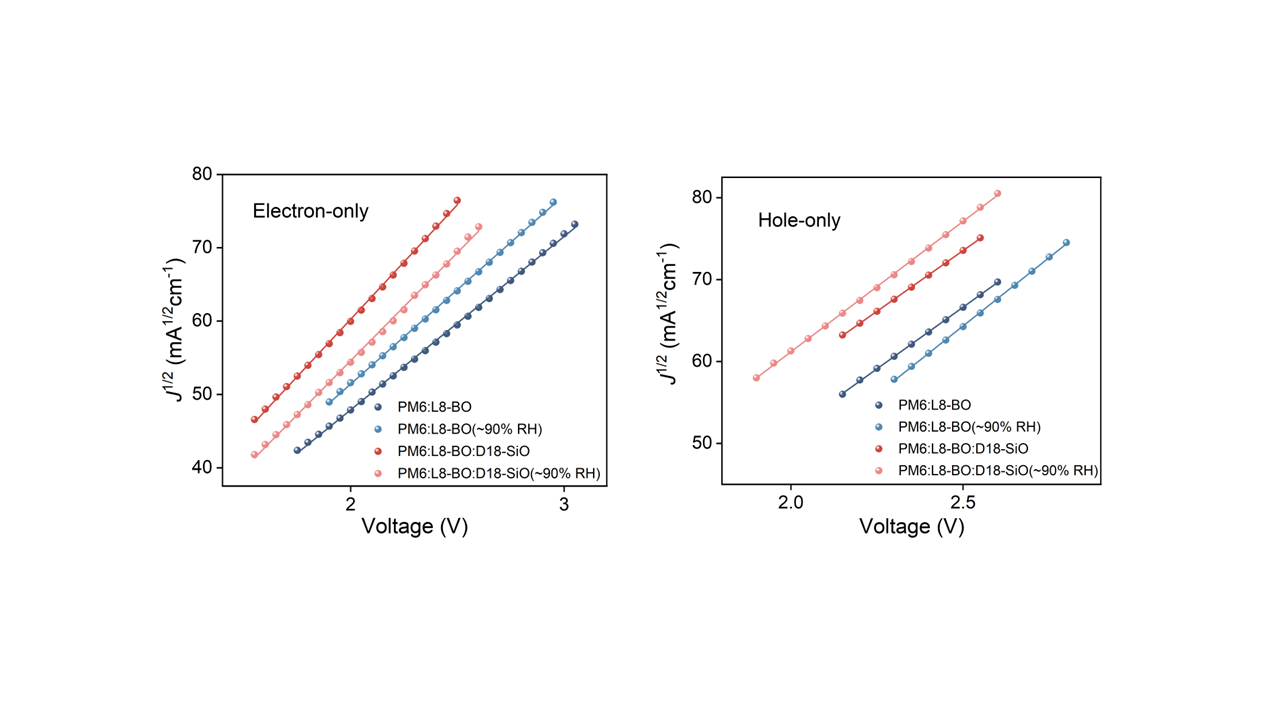


**Figure S14.** *J* ^1/2^ ~*V* fitting curves for hole mobilities and electron mobilities of the OSCs devices.


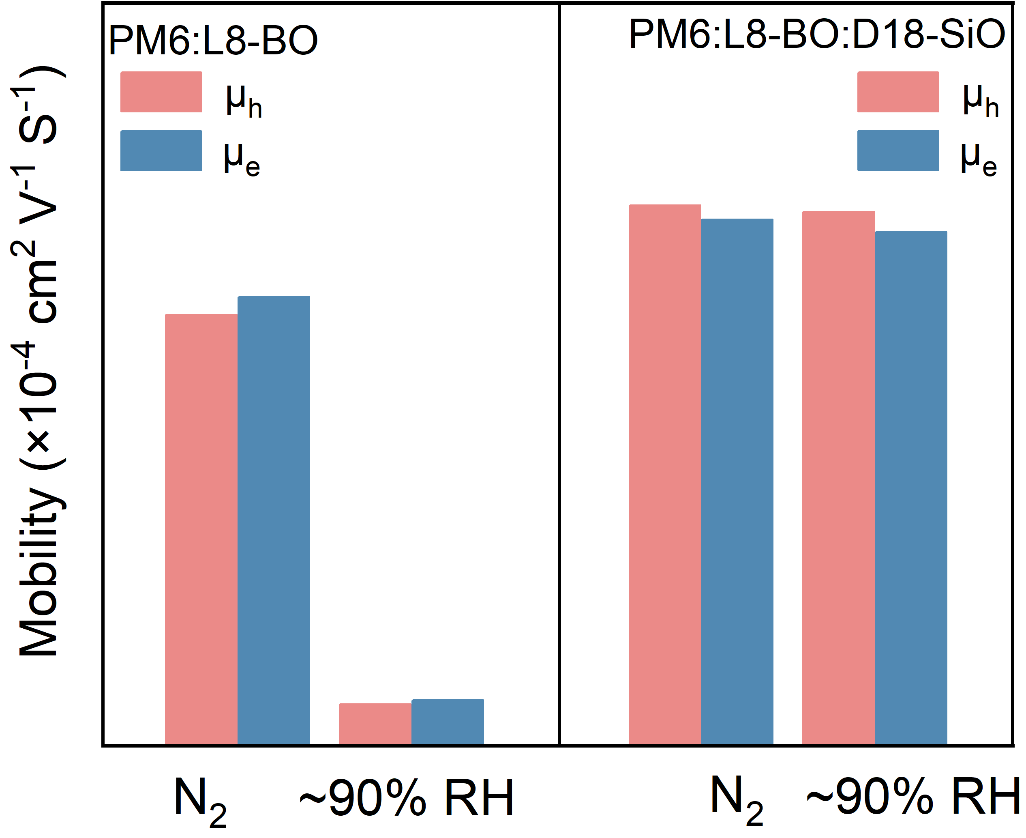


**Figure S15.** The hole and electron mobilities of the OSCs devices in different environment.

**
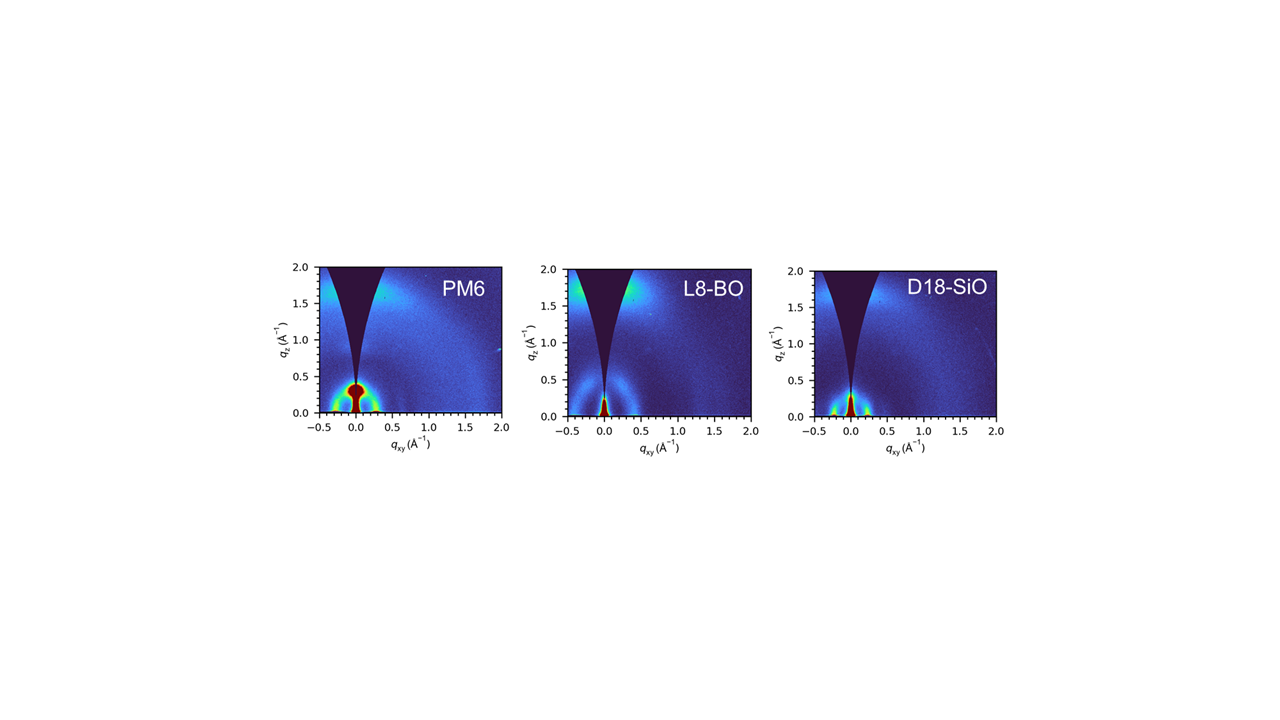
**

**Figure S16.** 2D-GIWAXS patterns for PM6, L8-BO and D18-SiO neat films.

**
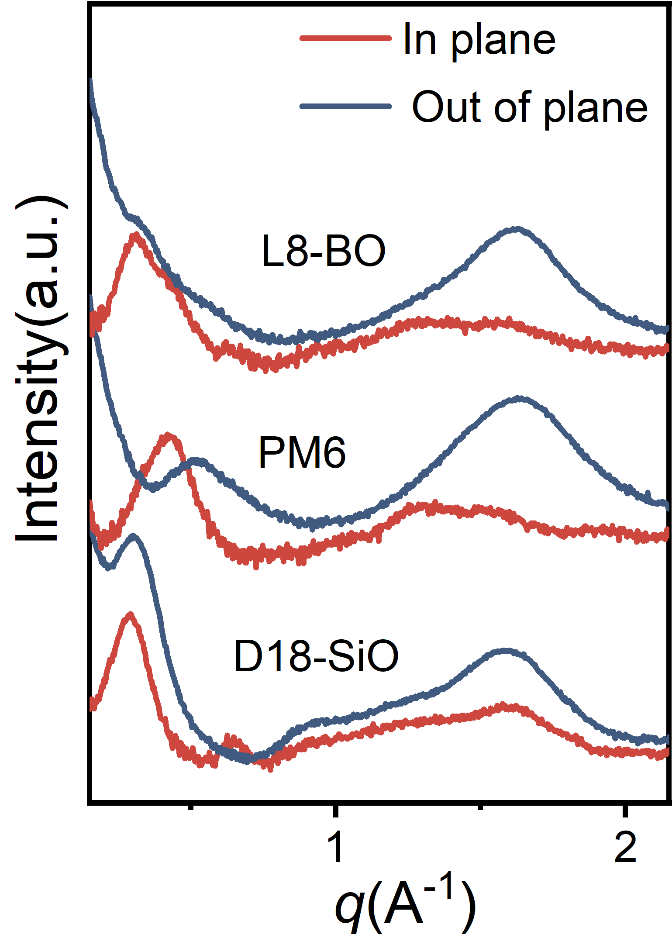
**

**Figure S17.** The in-plane (IP) and out-of-plane (OOP) extracted line-cut profiles of the corresponding pure films.

**
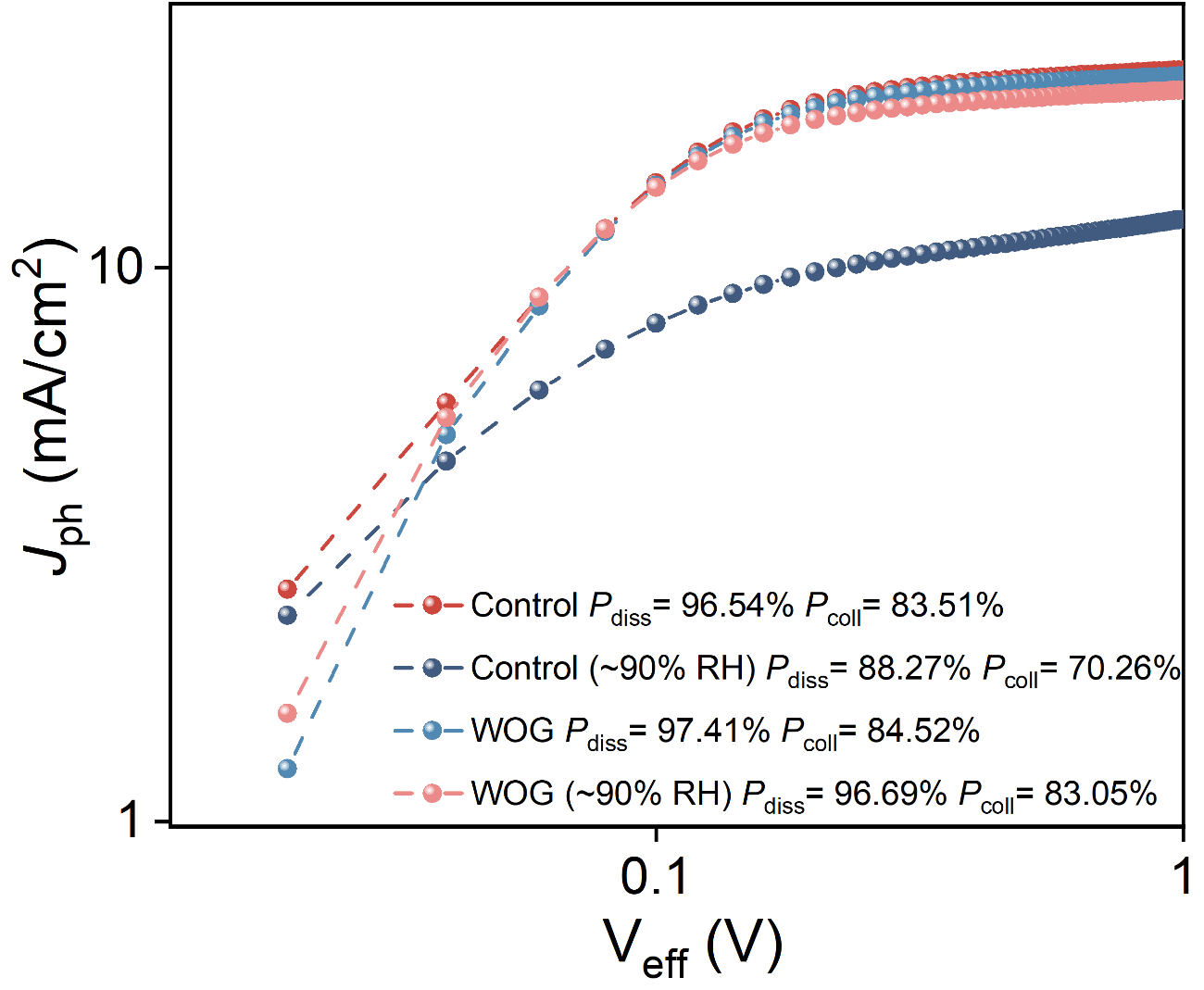
**

**Figure S18.** The photocurrent density (*J*_ph_) versus effective voltage (*V*_eff_) curves of the OSCs devices.


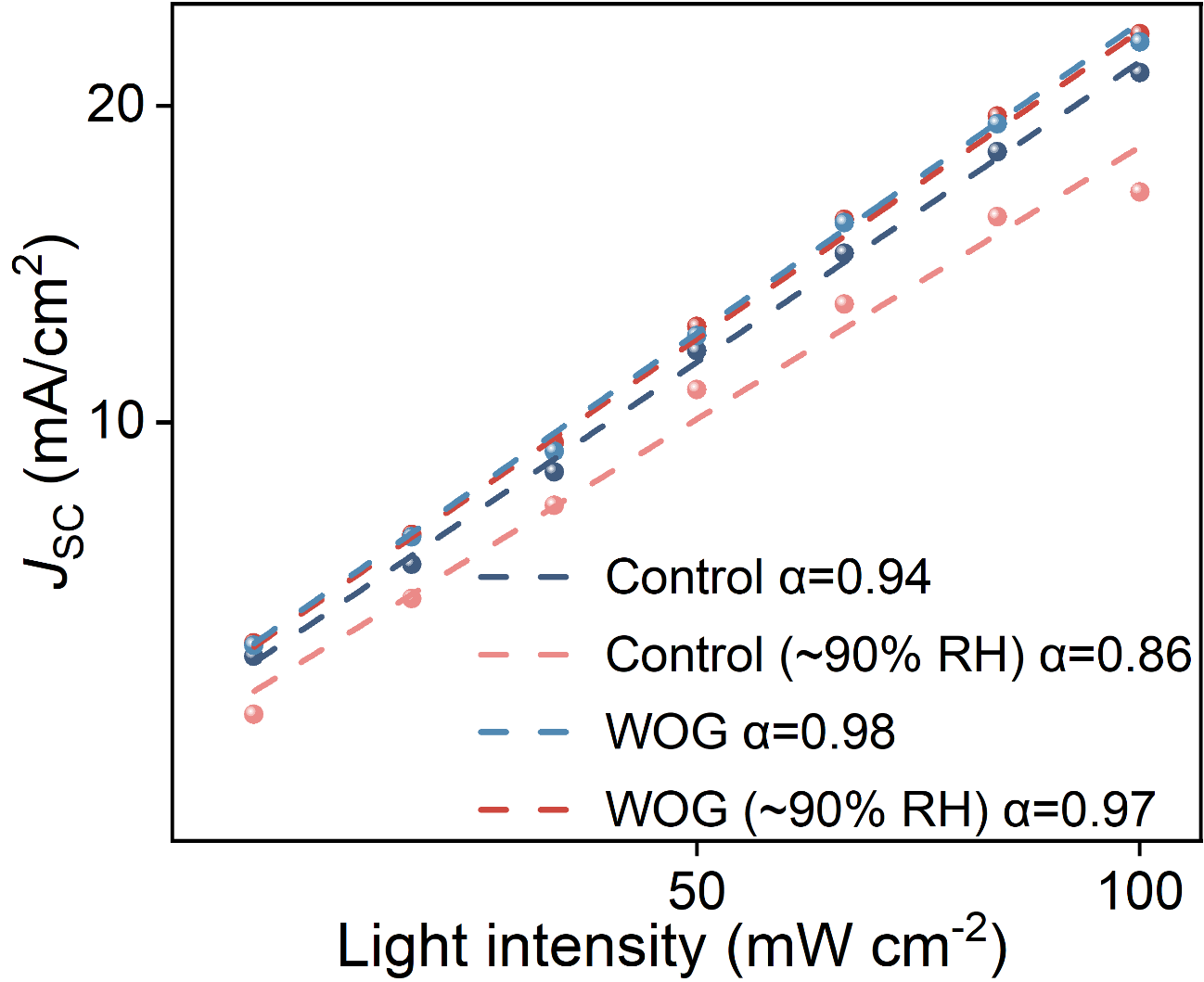


**Figure S19.** The dependence of *J*_SC_ on light intensity of the OSCs based on active layers.

**
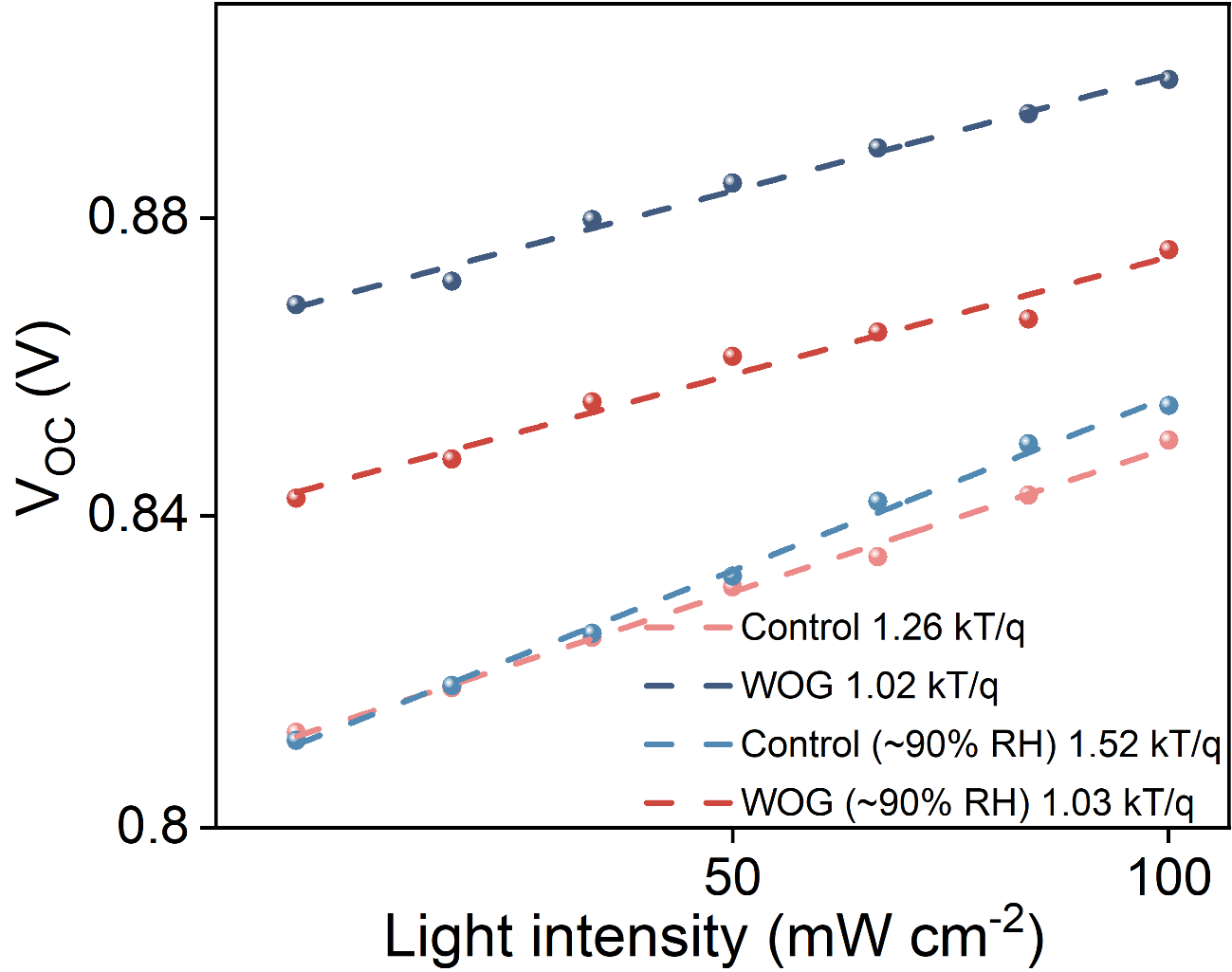
**

**Figure S20.** The dependence of *V*_OC_ on light intensity of the OSCs based on active layers.

**
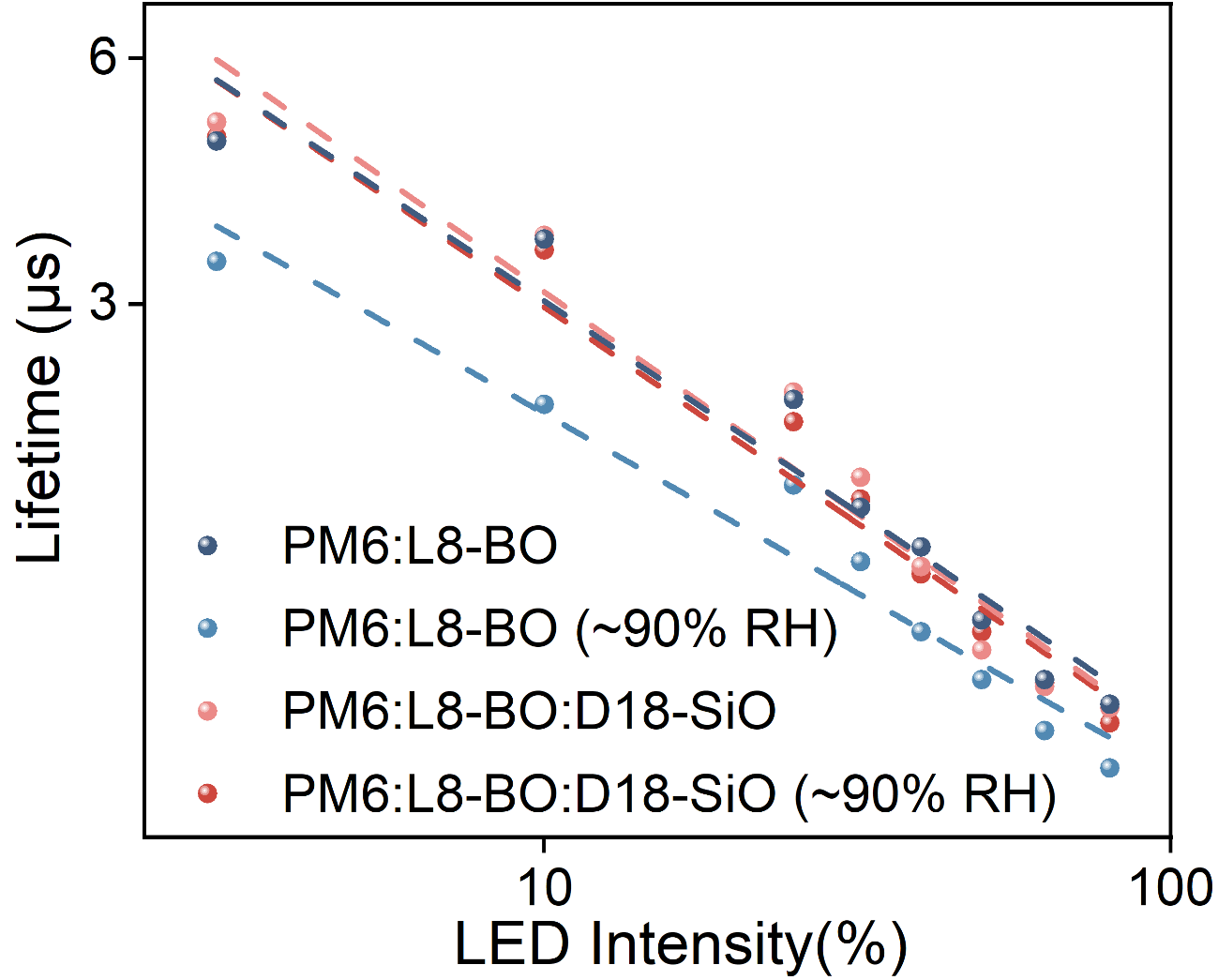
**

**Figure S21.** Carrier lifetime versus different illumination intensities of devices.

**
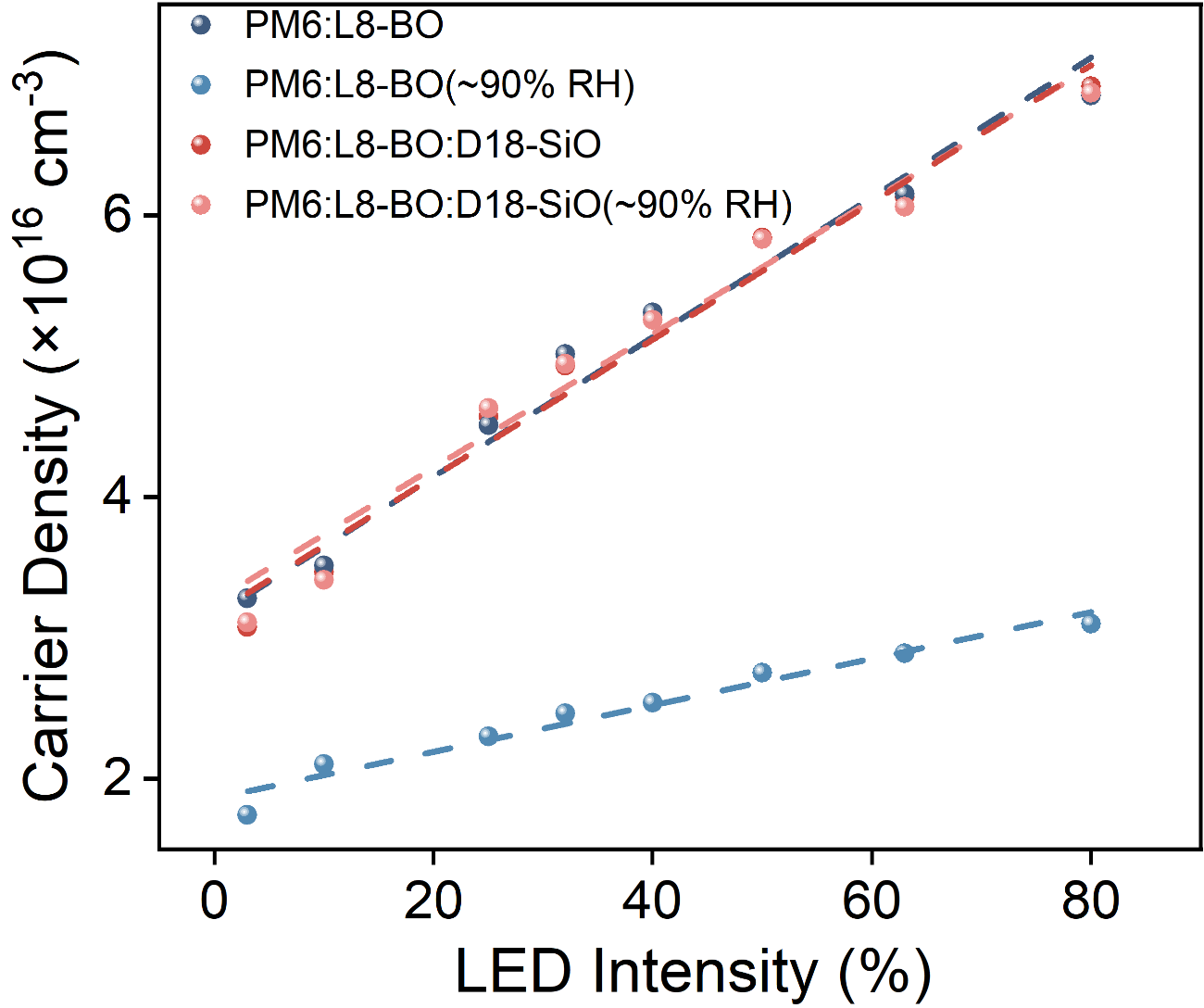
**

**Figure S22.** Carrier densities versus different illumination intensities of devices.


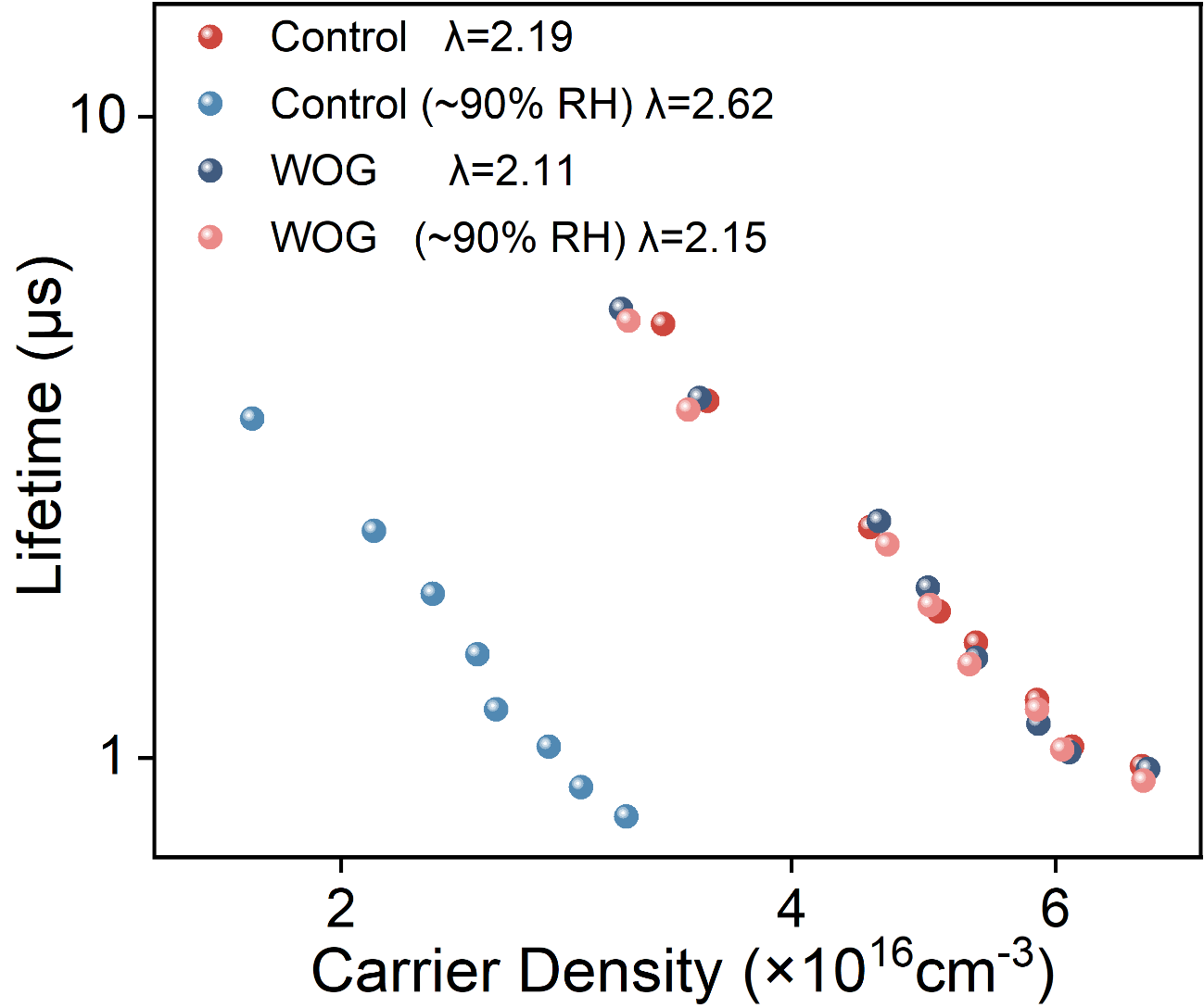


**Figure S23.** Carrier lifetime versus carrier density of Control and WOG devices under different environment.


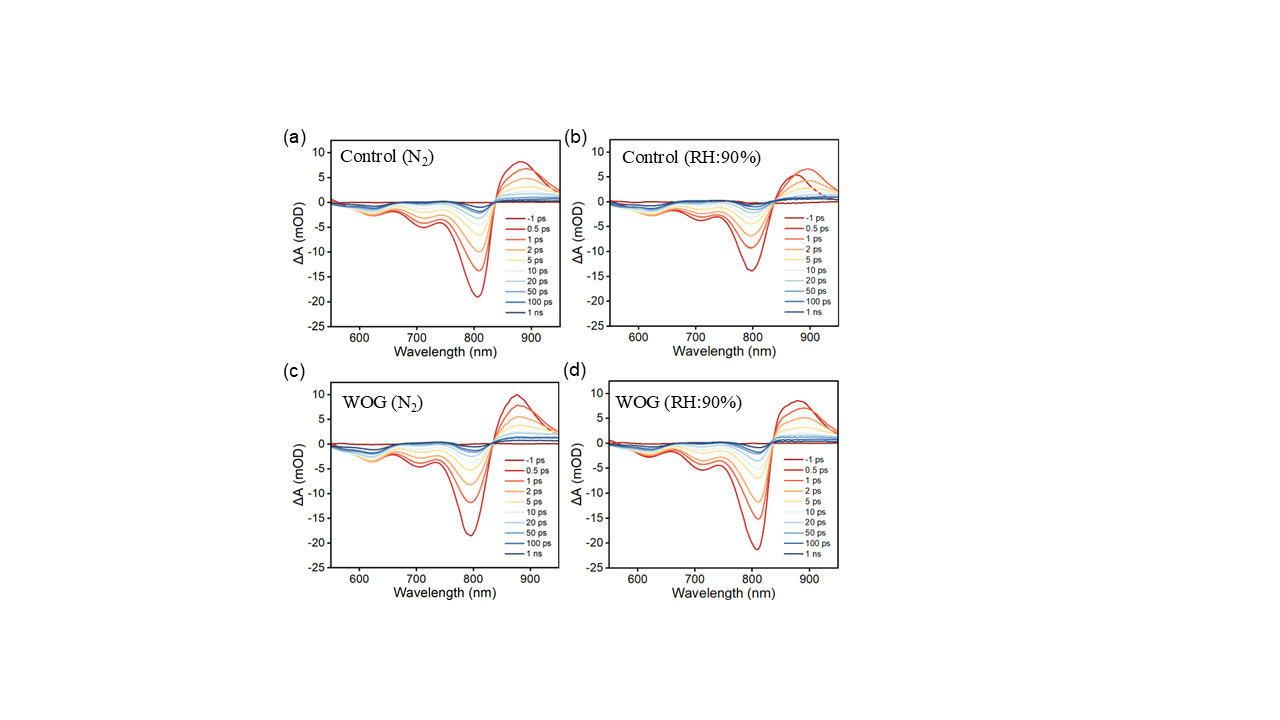


**Figure S24.** The representative spectra at indicated delay times of control and WOG films in different environment under 800 nm excitation.

**
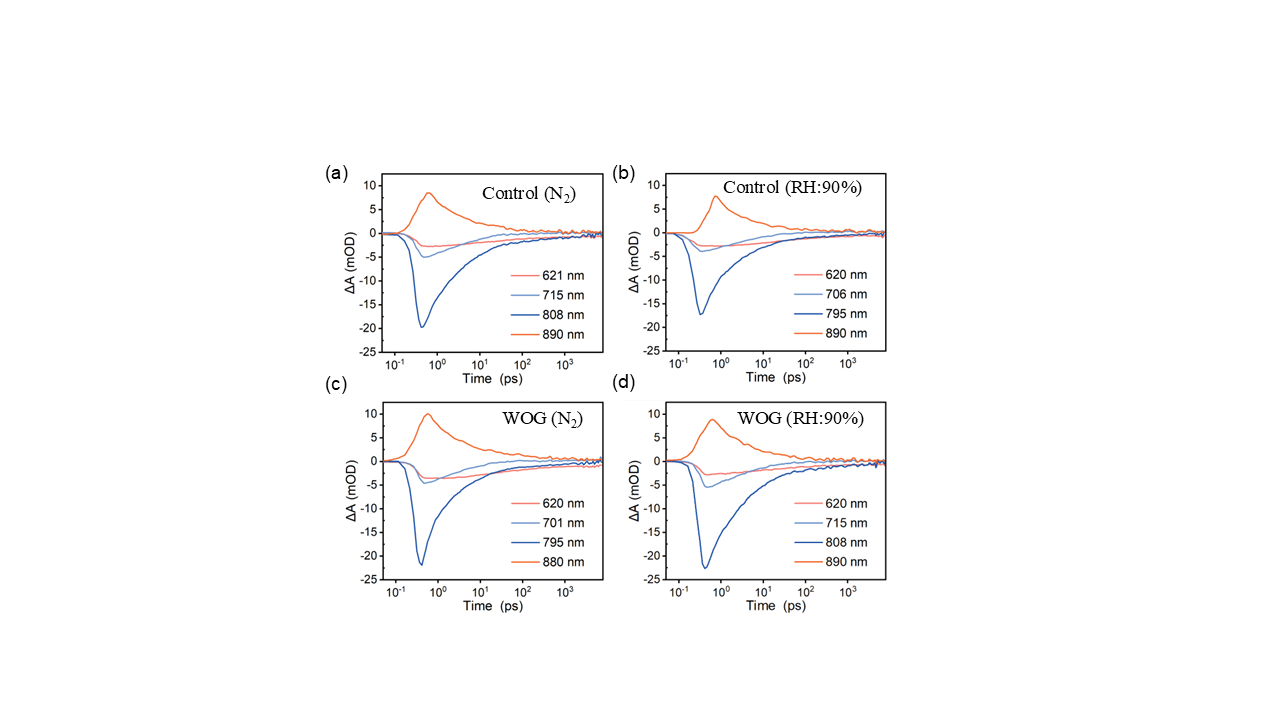
**

**Figure S25.** TA traces of control and WOG films in different environment.


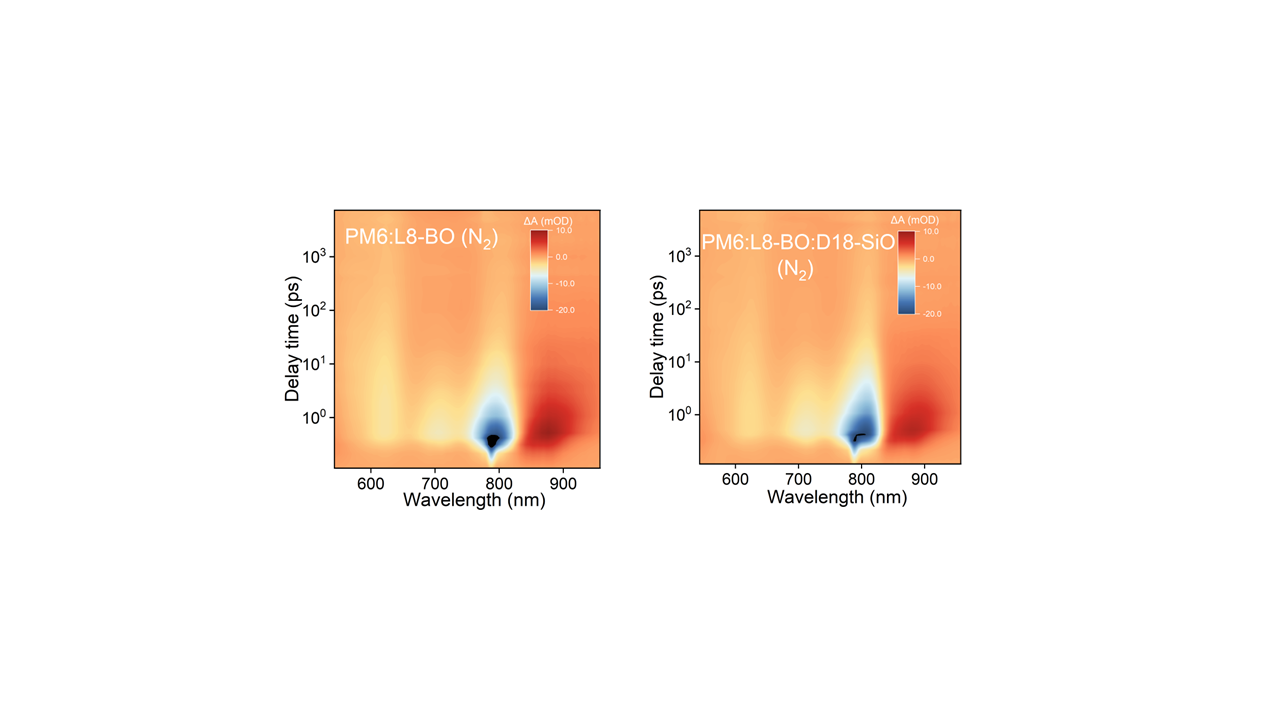


**Figure S26.** 2D color TA spectra of PM6: L8-BO and PM6: L8-BO: D18-SiO films.


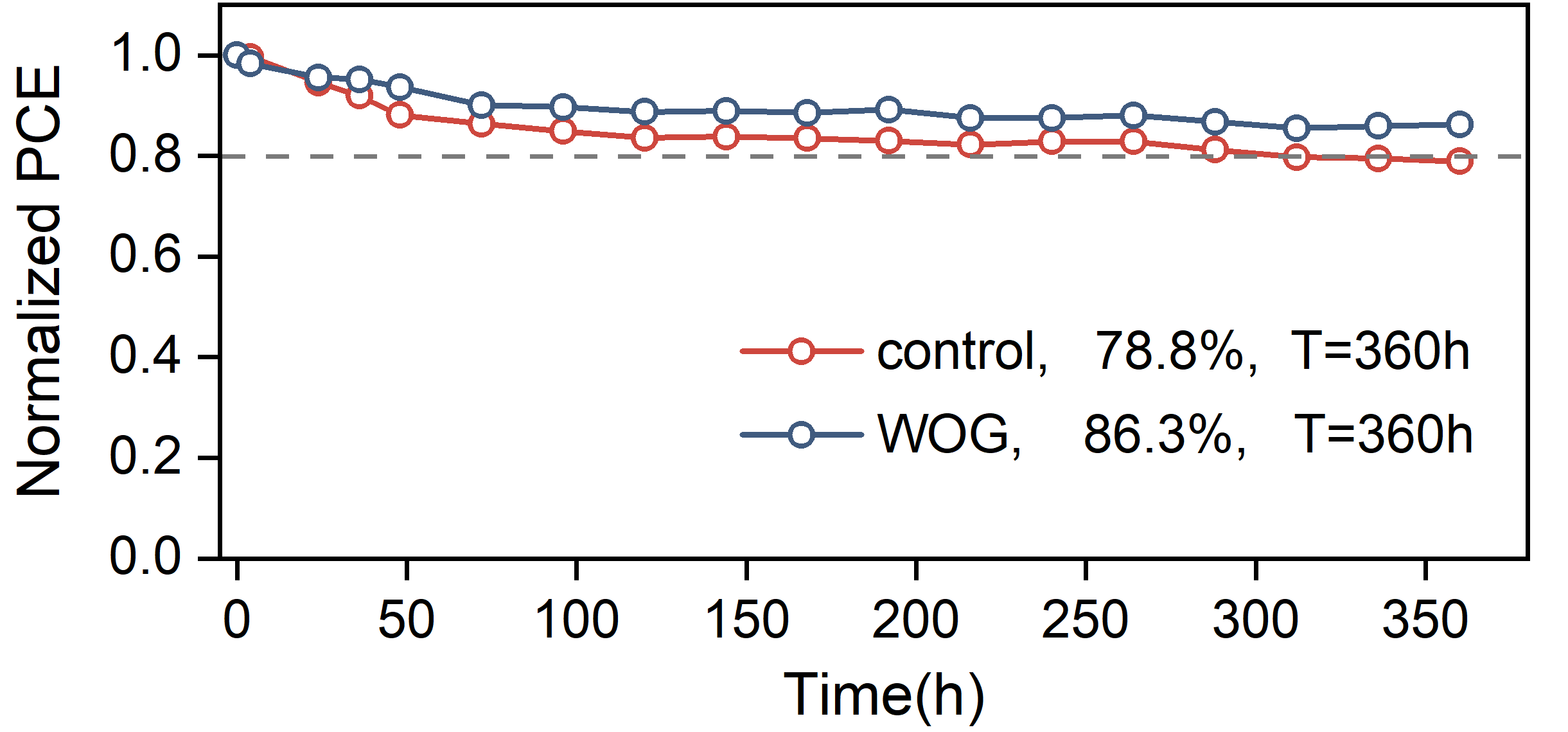


**Figure S27**. Thermal stability curve of inverted devices.


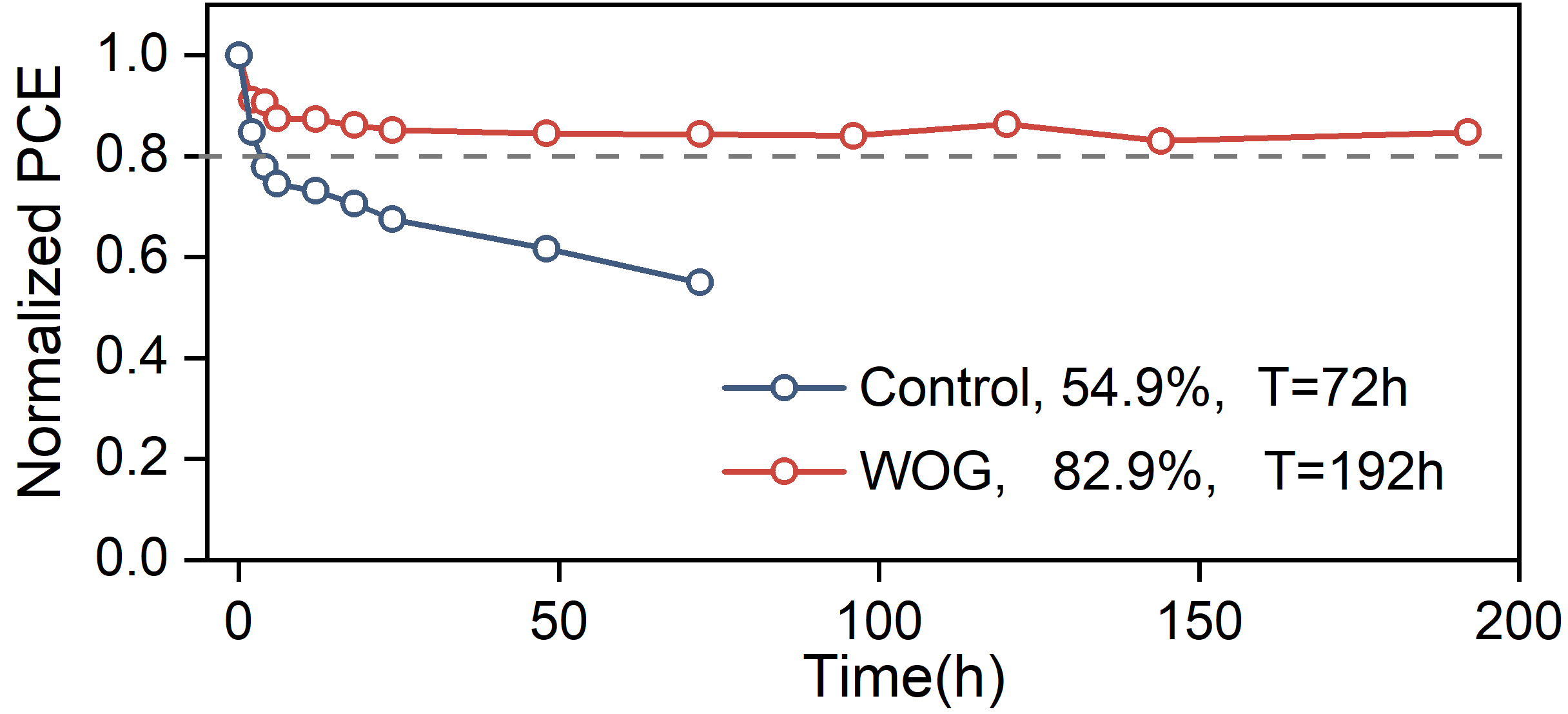


**Figure S28**. Thermal stability curve of conventional devices.


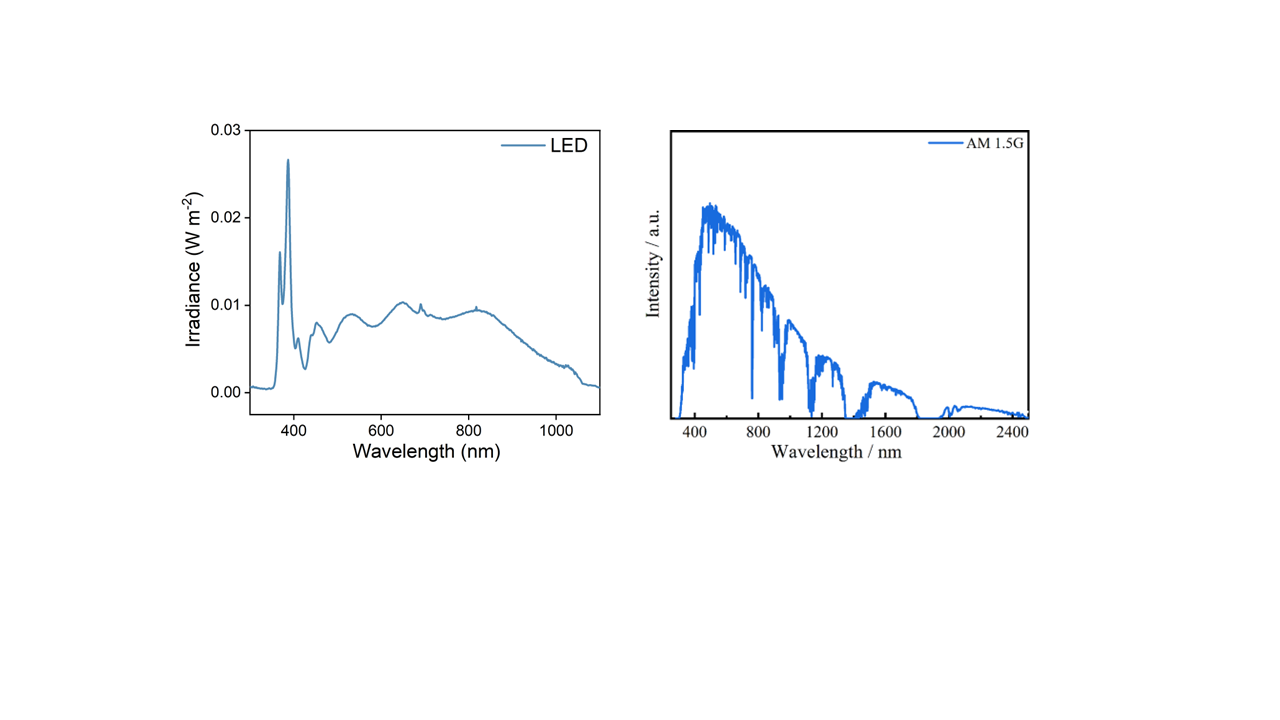


**Figure S29.** The spectral curves for both the LED and AM 1.5G sources.

**
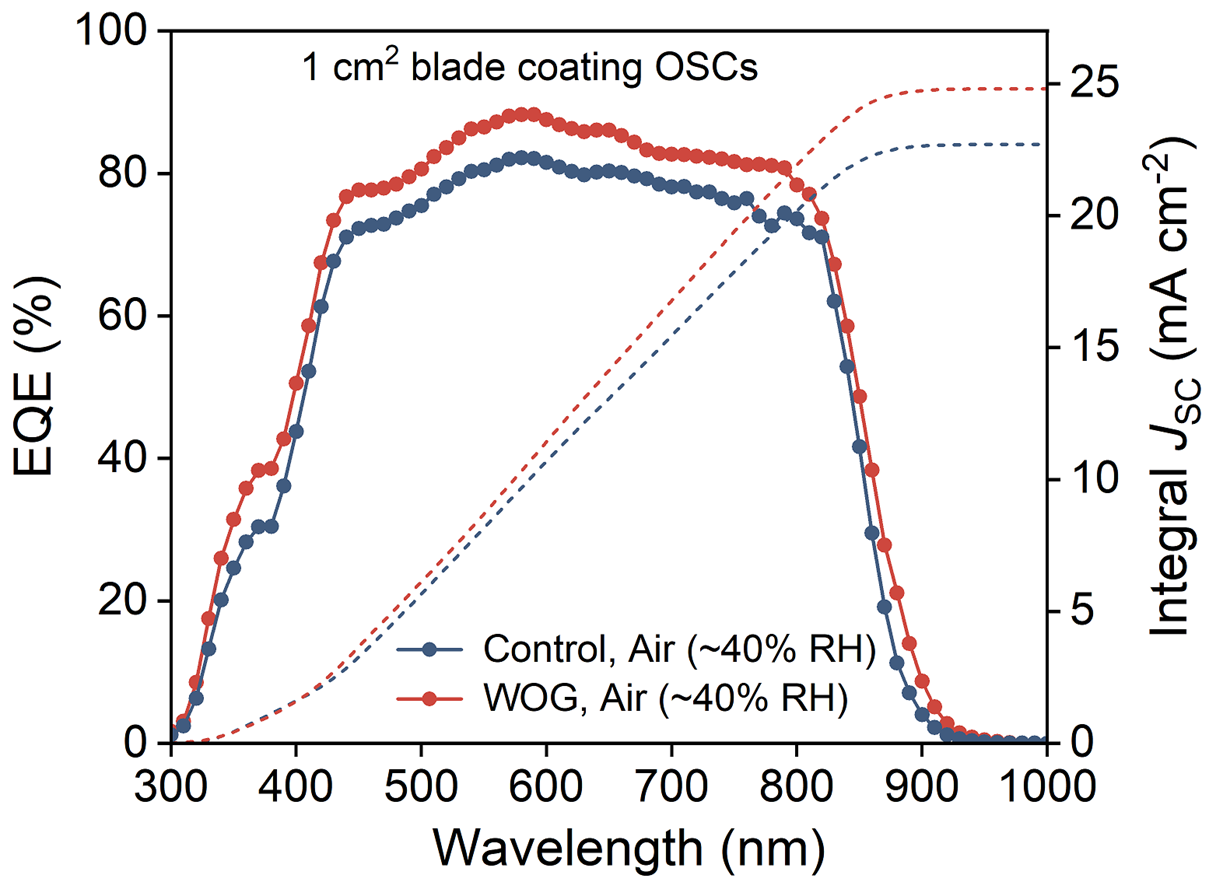
**

**Figure S30.** EQE curves of OSCs based on control and WOG active layer processed in air with 40% RH.


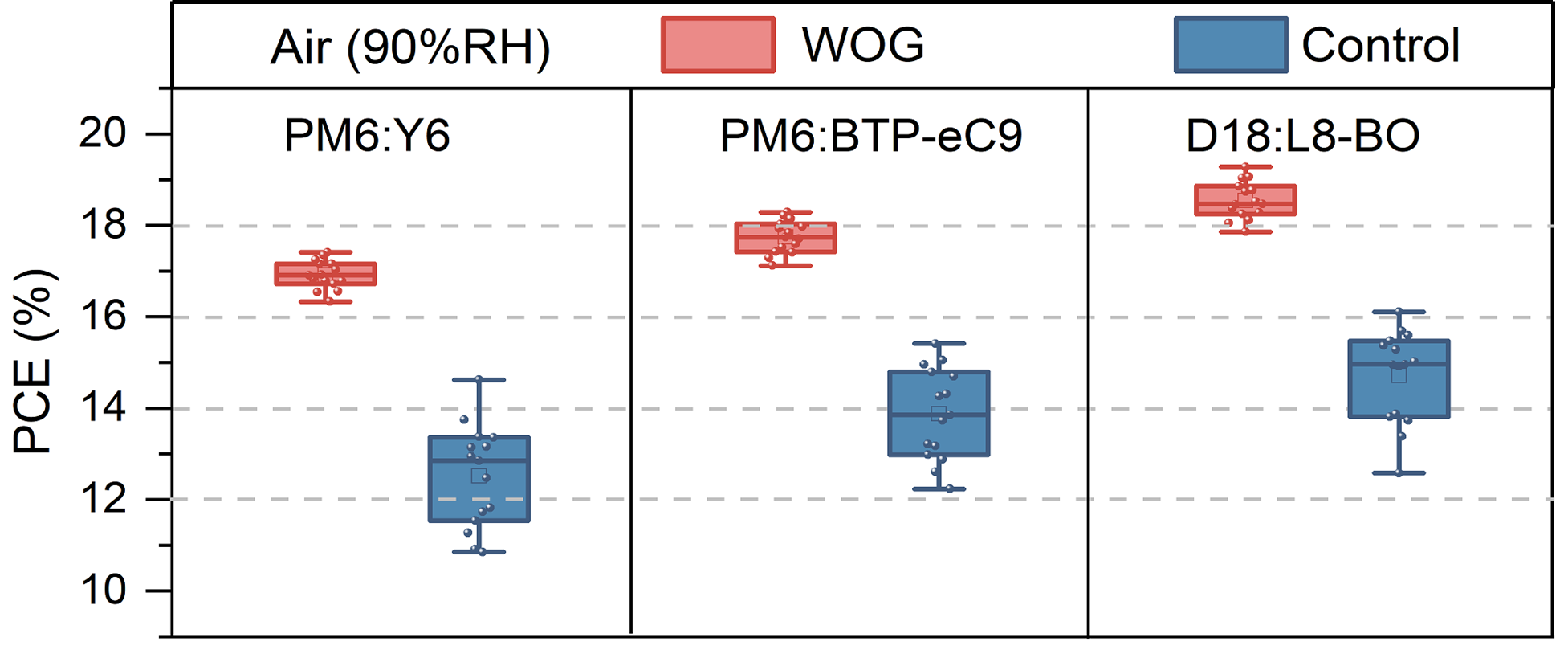


**Figure S31.** Repeatability of control and WOG devices under high humidity conditions.

**Table S1.** Detailed photovoltaic parameters of ternary system adding different proportion of D18-SiO without DIM treatment.

| Ratio  (D18-SiO%) | *V*_OC_  [V] | FF  [%] | *J*_SC_  [mA/cm^2^] | PCE  [%] |
| --- | --- | --- | --- | --- |
| 0% | 0.902 | 75.69 | 25.92 | 17.7 |
| 3% | 0.903 | 77.48 | 26.58 | 18.6 |
| 5% | 0.901 | 76.36 | 26.59 | 18.3 |
| 10% | 0.898 | 76.50 | 26.49 | 18.2 |

**Table S2.** Photovoltaic parameters of the inverted OSCs processed with and without WOG under the illumination of AM 1.5G (100 mW/cm^2^).

| Active layer (PM6:L8-BO) | Fabricating Condition | *V*_OC_  [V] | FF  [%] | *J*_SC_  [mA/cm^2^] | PCE  [%] |
| --- | --- | --- | --- | --- | --- |
| Control | N_2_ | 0.883 | 77.84 | 25.63 | 17.6 |
| Control | Air (RH:90%) | 0.872 | 74.82 | 24.88 | 16.2 |
| WOG | N_2_ | 0.894 | 78.39 | 25.93 | 18.1 |
| WOG | Air (RH:90%) | 0.892 | 78.74 | 26.21 | 18.4 |

**Table S3.** Summary of the Optical parameters of PM6, L8-BO, and D18-SiO.

| Sample | *λ*_max_ (nm) | | *λ*_onset_  (nm) | *E*_g_^opt^ [eV] |
| --- | --- | --- | --- | --- |
|  | solution | film |  |  |
| PM6 | 616 | 612 | 685 | 1.81 |
| L8-BO | 732 | 798 | 880 | 1.41 |
| D18-SiO | 569 | 580 | 637 | 1.95 |

**Table S4.** Contact angles and surface tensions of PM6, L8-BO and D18-SiO.

| Film | $\theta_{water}$(^o^) | $\theta_{oil}$(^o^) | $\text{γ}^{\text{d}}$(mJ m^-2^) | $\text{γ}^{\text{p}}$(mJ m^-2^) | $\text{γ}$(mJ m^-2^) |
| --- | --- | --- | --- | --- | --- |
| PM6 | 100.22 | 47.76 | 0.55 | 20.11 | 20.66 |
| L8-BO | 90.26 | 34.78 | 0.61 | 26.70 | 27.31 |
| D18-SiO | 108.27 | 55.41 | 0.52 | 15.66 | 16.18 |

**Table S5.** Contact angles and surface tensions for WOG-based active layer treated at 90% RH without and with PEDOT: PSS as the HTL.

| PEDOT: PSS | $\theta_{water}$(^o^) | $\theta_{oil}$(^o^) | $\text{γ}^{\text{d}}$(mJ m^-2^) | $\text{γ}^{\text{p}}$(mJ m^-2^) | $\text{γ}$(mJ m^-2^) |
| --- | --- | --- | --- | --- | --- |
| Without | 107.67 | 64.14 | 0.48 | 16.53 | 17.01 |
| With | 109.02 | 65.80 | 0.47 | 15.84 | 16.31 |

**Table S6.** Morphology data of donor and acceptor in the blend films.

| Sample | Out-of plane | | | In-plane | | |
| --- | --- | --- | --- | --- | --- | --- |
|  | *q*  (Å^-1^) | d-spacing (Å) | CCL (Å) | *q*  (Å^-1^) | d-spacing (Å) | CCL (Å) |
| PM6: L8-BO | 1.63 | 3.85 | 31.05 | 0.29 | 21.65 | 79.84 |
| PM6:D18-SiO: L8-BO | 1.64 | 3.82 | 46.57 | 0.30 | 20.93 | 111.78 |
| PM6: L8-BO (air) | 1.60 | 3.93 | 28.66 | 0.27 | 23.25 | 51.32 |
| PM6: D18-SiO: L8-BO (air) | 1.64 | 3.82 | 48.59 | 0.30 | 20.93 | 139.73 |

| Active layers | *μ*_h_  (10^−4^ cm^2^ V^−1^ s^−1^) | *μ*_e_  (10^−4^ cm^2^ V^−1^ s^−1^) | *μ*_h_ /*μ*_e_ |
| --- | --- | --- | --- |
| PM6: L8-BO (N_2_) | 4.06 | 4.23 | 0.96 |
| PM6: L8-BO (RH:90%) | 0.39 | 0.43 | 0.90 |
| PM6: D18-SiO: L8-BO (N_2_) | 5.09 | 4.96 | 1.02 |
| PM6: D18-SiO: L8-BO (RH:90%) | 5.03 | 4.84 | 1.03 |

**Table S7.** Summary of the fitting data for hole-only and electron-only device.

**Table S8.** Photovoltaic parameters of the blade coating 1 cm^2^ OSCs processed with and without WOG under the illumination of AM 1.5G (100 mW/cm^2^).

| Active layer | Fabricating  Condition | *V*_OC_[V] | FF [%] | *J*_SC_ [mA/cm^2^] ^a)^ | PCE [%] |
| --- | --- | --- | --- | --- | --- |
| PM6:L8-BO | Blade coating, Air (~40% RH) | 0.845 | 72.5 | 24.5 | 15.1 |
| PM6: D18-SiO: L8-BO | Blade coating, Air (~40% RH) | 0.869 | 76.2 | 26.1 | 17.3 |

^a)^ The *J*_cal_ represents the integrated current density obtained from EQE spectra.

**Table S9.** *J*-*V* of the different photovoltaic systems processed with and without WOG at ~90% RH.

| Active layer | Fabricating Condition | *V*_OC_[V] | FF [%] | *J*_SC_ [mA/cm^2^] ^a)^ | PCE [%] |
| --- | --- | --- | --- | --- | --- |
| PM6:Y6 | Air (~90% RH) | 0.812 | 70.8 | 25.4 | 14.6 |
| PM6: Y6: D18-SiO | Air (~90% RH) | 0.846 | 76.6 | 26.8 | 17.4 |
| PM6: BTP-eC9 | Air (~90% RH) | 0.817 | 73.8 | 25.5 | 15.4 |
| PM6: BTP-eC9: D18-SiO | Air (~90% RH) | 0.854 | 78.8 | 27.2 | 18.3 |
| D18:L8-BO | Air (~90% RH) | 0.869 | 73.4 | 25.2 | 16.1 |
| D18: L8-BO: D18-SiO | Air (~90% RH) | 0.913 | 80.1 | 26.4 | 19.3 |

^a)^ The *J*_cal_ represents the integrated current density obtained from EQE spectra.

**NMR Spectrum**

**
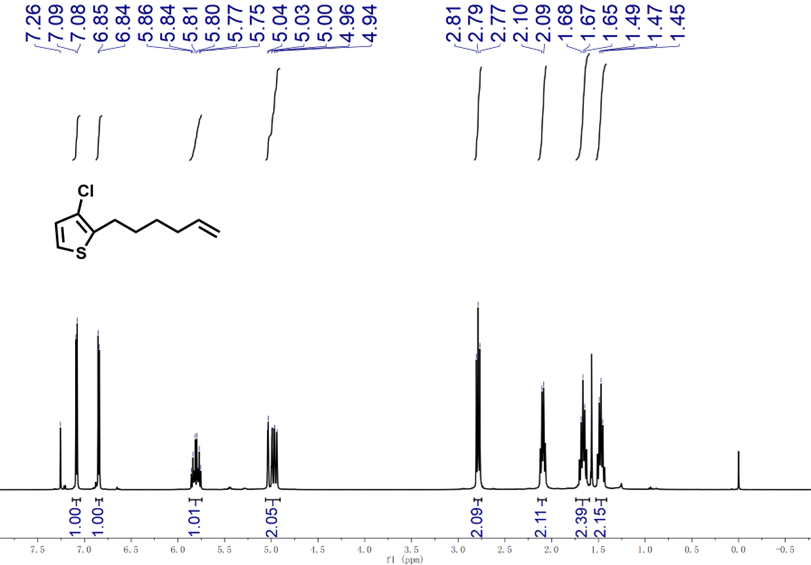
**

**Figure S32.** ^1^H NMR spectrum of compound 1.

**
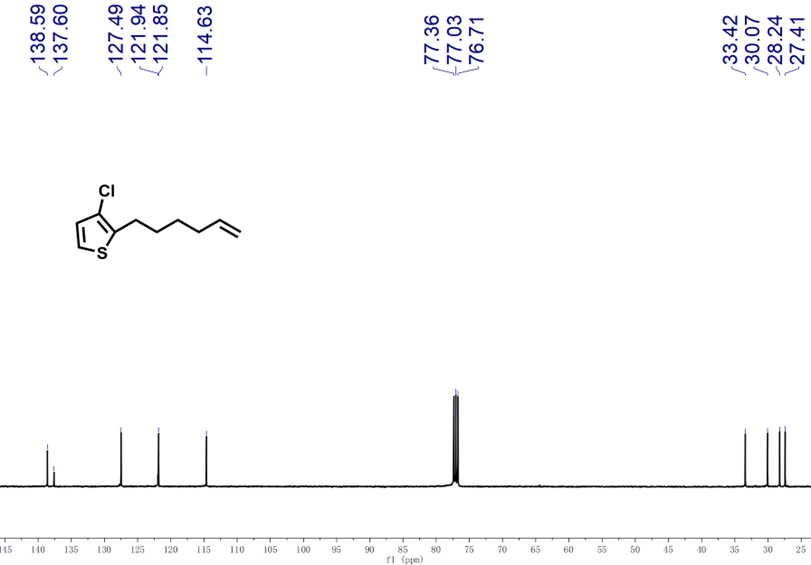
**

**Figure S33.** ^13^C NMR spectrum of compound 1.

**
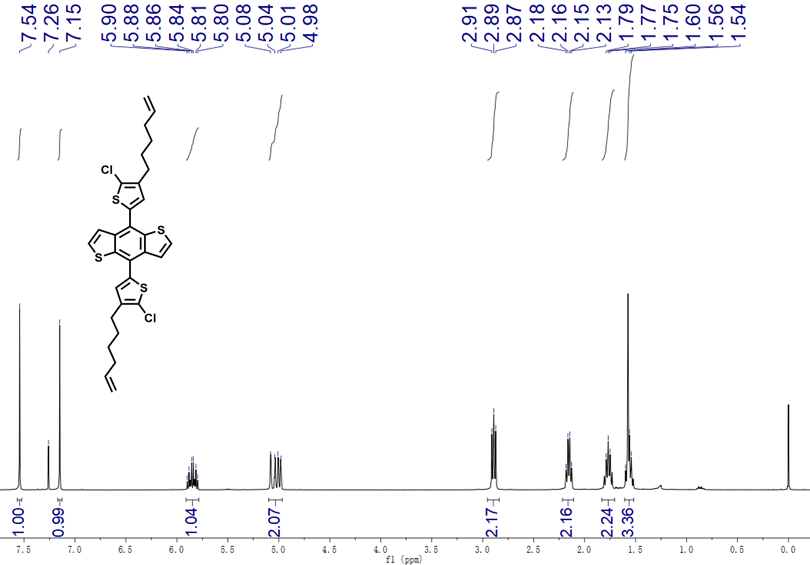
**

**Figure S34.** ^1^H NMR spectrum of compound 2.

**
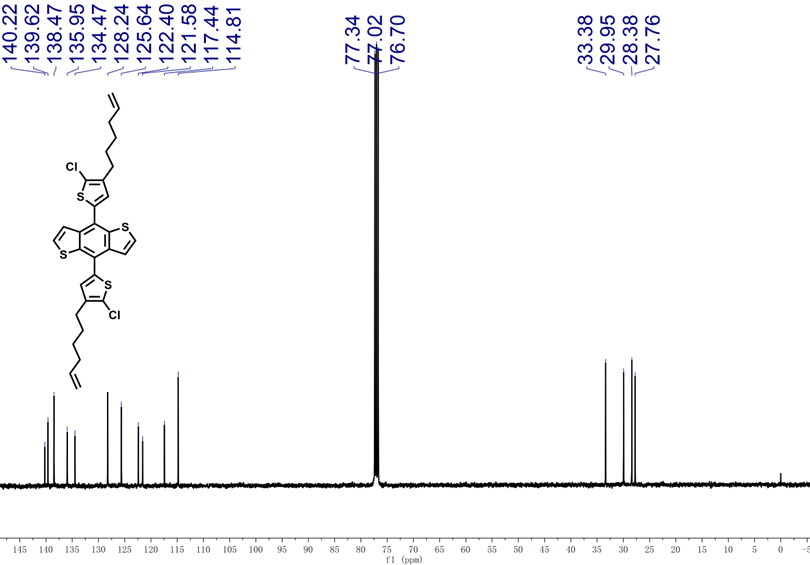
**

**Figure S35.** ^13^C NMR spectrum of compound 2.

**
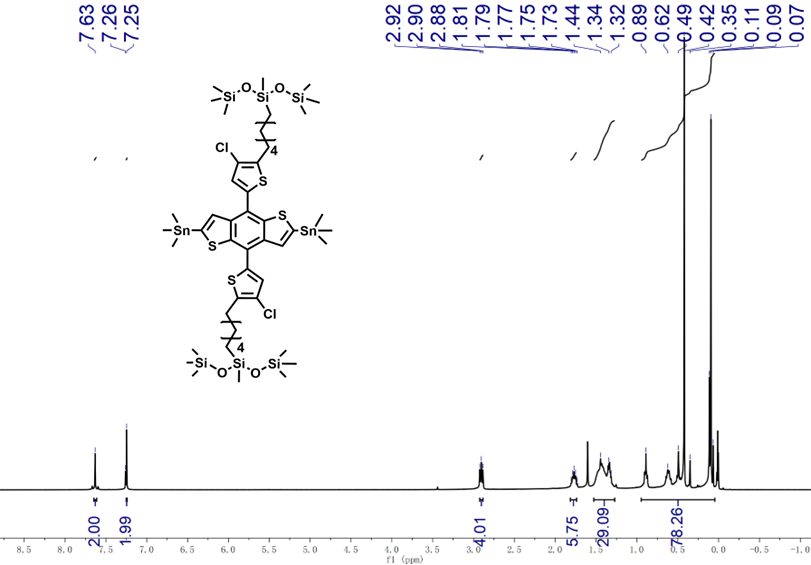
**

**Figure S36.** ^1^H NMR spectrum of compound 4.


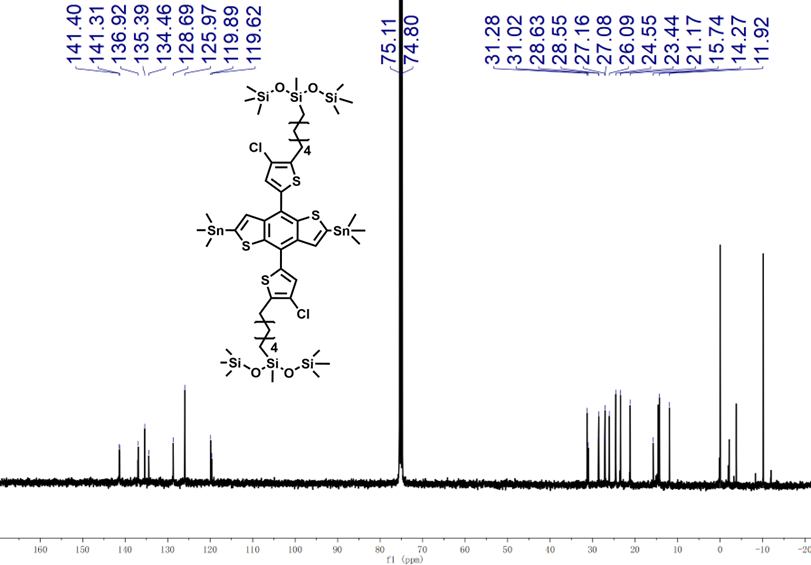


**Figure S37.** ^13^C NMR spectrum of compound 4.

**Reference**

[1] Q. Wang, X. Zhang, W. Zou, H. Xu, Y. Kan, Y. Sun, K. Gao, *Chin. J. Chem.* **2023**, 41, 3703.

[2] H. Liu, D. Yuan, H. Jiang, S. Li, L. Zhang, J. Chen, *Energy Environ. Sci.* **2023**, 16, 3474.

[3] X. Chen, M. Chen, J. Liang, H. Liu, X. Xie, L. Zhang, D. Ma, J. Chen, *Adv. Mater.* **2024**, 36, 2313074.

[4] T. Lu, Q. Chen, *Comput. Theor. Chem.* **2021**, 1200,113249.

[5] L. Martínez, R. Andrade, E. G. Birgin, J. M. Martínez, *J. Comput. Chem.* **2009**, 30, 2157.

[6] M. Schauperl, P. S. Nerenberg, H. Jang, L.-P. Wang, C. I. Bayly, D. L. Mobley, M. K. Gilson, *Commun. Chem.* **2020**, 3,44.

[7] C. Liu, Y. Fu, J. Zhou, L. Wang, C. Guo, J. Cheng, W. Sun, C. Chen, J. Zhou, D. Liu, W. Li, T. Wang, *Adv. Mater.* **2023**, 36, 2308608.

[8] J. Wang, L. Spada, J. Chen, S. Gao, S. Alessandrini, G. Feng, C. Puzzarini, Q. Gou, J. U. Grabow, V. Barone, *Angew. Chem. Int.Edit.* **2019**, 58, 13935.

[9] R. Di Pietro, D. Fazzi, T. B. Kehoe, H. Sirringhaus, *J. Am. Chem. Soc.* **2012**, 134, 14877.

[10] Z. Gan, L. Wang, J. Cai, C. Guo, C. Chen, D. Li, Y. Fu, B. Zhou, Y. Sun, C. Liu, J. Zhou, D. Liu, W. Li, T. Wang, *Nat. Commun.* **2023**, 14, 6297.

[11] Y. Wang, S. D. Motta, F. Negri, R. Friedlein, *J. Am. Chem. Soc.* **2011**, 133, 10054.
